# Density functional theory investigation of mechanisms of degradation reactions of sulfonated PEEK membranes with OH radicals in fuel cells: addition–elimination reactions and acid catalyzed water elimination

**DOI:** 10.1007/s00214-023-02981-2

**Published:** 2023-04-25

**Authors:** Jonathan E. Stevens, Courtney M. Pefley, Alice Piatkowski, Zachary R. Smith, Nikolina Ognanovich

**Affiliations:** 1 Department of Chemistry and Biochemistry, University of Detroit Mercy, Detroit, MI 48221 USA; 2 School of Dentistry, University of Detroit Mercy, Detroit, MI 48208 USA; 3 American Axle Manufacturing, Detroit, MI 48221 USA; 4 School of Public Health, University of Michigan, Ann Arbor, MI 48109 USA

**Keywords:** Density functional theory, M062X, Fuel cell membrane, sPEEK, Hydroxy radical

## Abstract

Sulfonated polyether (ether) ketone or sulfonated PEEK (sPEEK) membranes are one possible candidate for proton-transfer membranes in hydrogen fuel cells. Reaction with hydroxy radicals is expected to be a significant source of degradation of these membranes during fuel cell operation. In this work, the reactivity of the sPEEK polymer molecule with OH radicals is studied by M062X hybrid density functional calculations of the energetics of several reaction paths in a water environment as modeled by polarized continuum model calculations. Reactants, products, encounter minima and transition states are optimized for a reaction pathway in which OH addition is followed by acid-catalyzed water elimination which cationizes the polymer, degradation is expected to follow this reaction as the unstable cation then undergoes bond-breaking or other reactions. Two pathways for this acid-catalyzed cationization, one in which a water molecule plays the role of an additional co-catalyst, are reported. Further calculations explore reaction pathways in which addition of OH to the polymer is followed by bond breaking reactions which would break the polymer chain or the bond between the polymer and sulfonyl groups. Examination of the free energy barriers to all these reactions, relative to reactants, suggests that these direct bond-breaking reactions may compete somewhat with acid-catalyzed water elimination following OH addition.

**Supplementary Information:**

The online version contains supplementary material available at 10.1007/s00214-023-02981-2.

## Introduction

Fuel cells of particular interest for automotive applications are proton-exchange membrane fuel cells (PEMFCs) [1, 2]. A key component of such devices is the proton exchange membrane (PEM) [1], a semipermeable membrane which allows protons produced at the anode to migrate through to the cathode of the fuel cells [2]. Currently, proton exchange membranes are typically perfluoroalkylsulfonic acid (PFSA) membranes [1, 2], especially Nafion [2–5]. However, fluorinated membranes and fluorinated polymers in general present environmental hazards upon degradation [6–8].

**Fig. 1:**
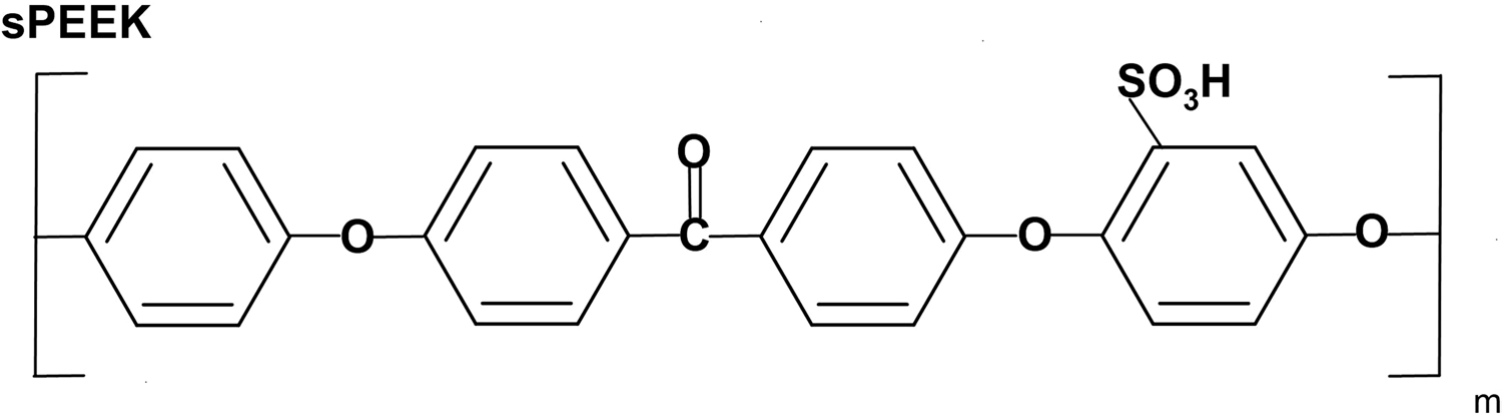
Repeating unit of SPEEK polymer

One of several proposed non-fluorinated alternatives to PFSA membranes is sulfonated aromatic membranes, such as the polymer sulfonated polyether(ether) ketone, or sulfonated PEEK (sPEEK) [2, 6, 7], displayed in Fig. 1.

Both PFSA and sulfonated aromatic membranes such as sPEEK are expected to be subject to degradation during fuel cell operation through reaction with OH [1, 7, 9, 10], H [1, 6, 9, 11], and OOH [1, 9, 11] radicals or H_2_O_2_ [12, 13] molecules. In the case of Nafion this degradation has been studied experimentally [9, 12, 14, 15] and there have also been recent computational works on the degradation reactions of Nafion with OH and H radicals [5, 16, 17] with H_2_O_2_ [12], and most recently with a hypothesized H_3_O radical [18].

The formation of OH radicals within a fuel cell, as stated in references [1, 5, 9], is believed to occur as a result of reactions resulting from the presence of O_2_, H_2_, and a platinum metal catalyst. Reference [8] notes that H_2_O_2_ may be formed from a reaction of O_2_ and H^+^ at the cathode, and that H_2_O_2_ molecules could react at the metal surface, or possibly on traces of transition metal cations, to produce two OH radicals.

Reference [5] presents computations of the barrier and energy of the reaction of H_2_O_2_ on a platinum catalyst surface to form OH radicals. In reference [5], the decomposition of H_2_O_2_ to form OH radicals is considered as one step in a multi-step mechanism for the production of OH radicals from reactions of H_2_ and O_2_ gases at the metal surface. This mechanism and a similar mechanism involving the production of an OOH intermediate (as opposed to H_2_O_2_) are noted to have low energetic barriers. The authors of reference [5] conclude that the metal surface plays an important role in production of OH radicals within fuel cells.

The degradation of sPEEK in the presence of OH radicals is expected to begin with the formation of an adduct of OH with aromatic rings within the polymer. Reference [1] notes that the rate of reaction of OH radicals with aromatic rings is extremely high, and estimates that the rate of OH reaction with the aromatic rings of sPEEK in a water-swollen coiled polymer would be orders of magnitude higher than the unzipping reactions of OH radicals in water-swollen Nafion. Reference [1] thus states that the aromatic rings will be subject to OH attack. Following formation of the adduct, an acid catalyzed water elimination reaction is expected to occur. This process is expected to proceed through of OH addition to the aromatic ring, followed by a protonation of the OH adduct, then elimination of water to leave a cationized aromatic ring [1, 10, 19, 20]. This degradation process for aromatic rings has been hypothesized by experimentalists attempting to study the degradation of sulfonated polymers in the presence of OH with EPR methods. Huber and Roduner [10] studied the reactions of a number of sulfonated aromatic model molecules with OH radicals. EPR experiments observe the formation of benzyl radicals in aqueous solution in the presence of acid. Reference [10] suggests this phenomenon initiates from an attachment of OH to the aromatic ring, followed by protonation, then followed by elimination of a water molecule to leave an aromatic cation. The benzyl radical is then formed by elimination of a proton from a methyl group attached to the aromatic ring.

This proposed mechanism is consistent with a mechanism reported for the production of benzyl radicals in reactions of toluene [19] and other methylbenzenes [20]. In the case of sPEEK, this process is expected to cationize the membrane [1]. In the case of sPEEK, there are no methyl groups attached to the aromatic rings, so the formation of benzyl radicals is not anticipated. Reference [1] states that cationization of the membrane is expected to lead to chain-breaking, cross-linking, or further hydroxylation reactions.

More recent experiments have used spin-trapping methods to explore the reactions of sPEEK with OH directly both in the in situ environment of an operating sPEEK-membrane hydrogen fuel cell [11] and in ex situ [21] experiments. Ex situ experiments found evidence of membrane degradation, detecting both phenoxy and phenyl radical products from sPEEK exposure to OH. In situ experiments observed no membrane degradation products; in reference [21], the authors note that run times for in situ experiments were short, and suggest such phenoxy and phenyl radical products would appear if longer fuel cell run times had been used.

Computational investigation of the degradation reactions of sPEEK in the presence of OH radicals has been reported in two works. Panchenko [7] has examined the thermodynamic feasibility of some reactions of non-fluorinated sulfonated aromatic membranes with OH radicals, focusing on model molecules for the polymers sPEEK and PSU (polyethersulfone). The calculations studied the thermodynamics of two types of reactions: abstraction of H atoms in sPEEK and PSU by OH groups, and also on the attachment of OH groups to some of the carbon atoms on the aromatic rings present, followed by reactions with O_2_ molecules. This work implemented the B3LYP functional with 6-31G(*d*) and 6-311 + *G*(*d*, *p*) basis sets and used the polarizable continuum model (PCM) to incorporate effects of solvation in water. In some cases, sulfonic acid groups were modeled in both protonated and deprotonated form.

**Fig. 2:**
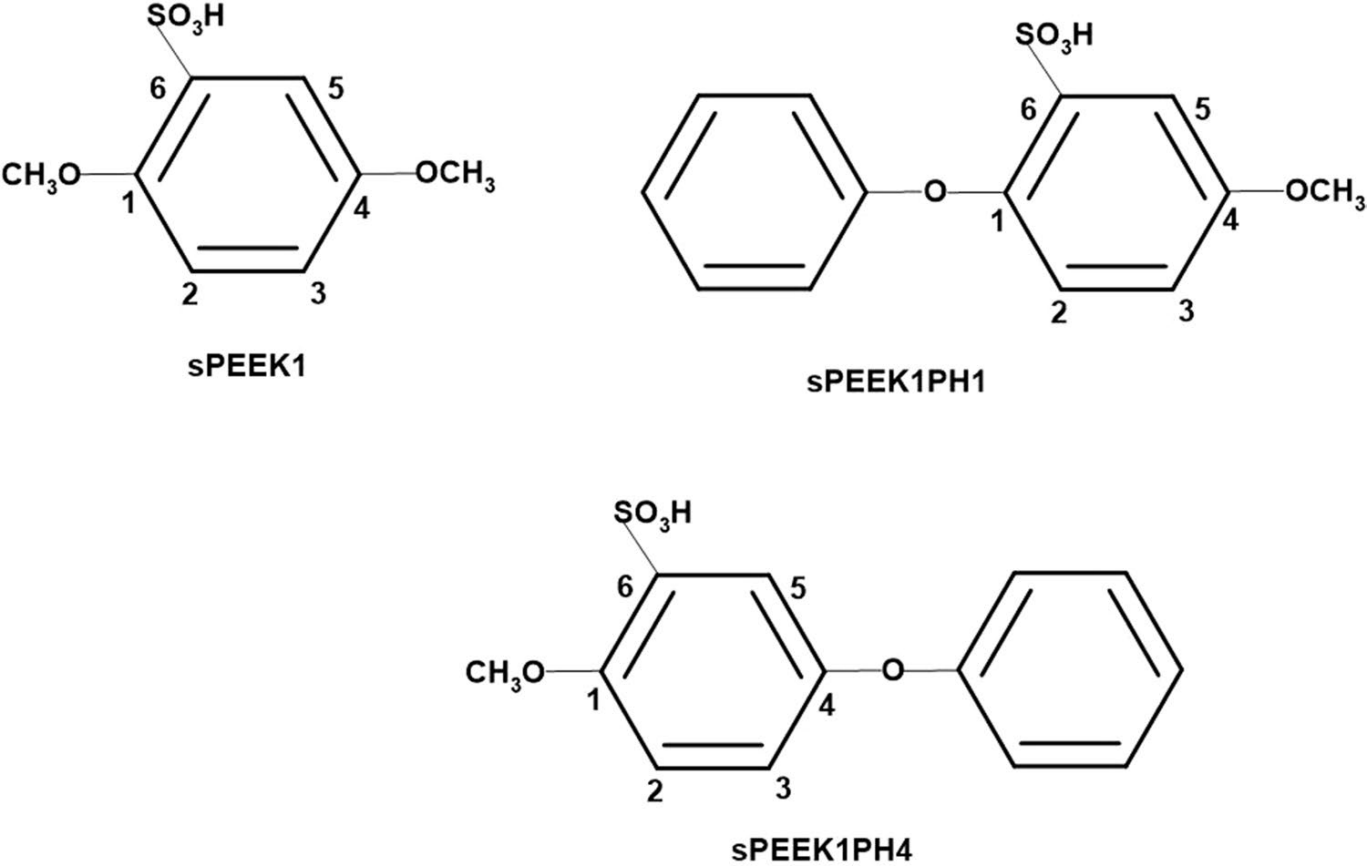
Model molecules for sPEEK

The second work [6] studied the reactions of sPEEK with H radicals in a solvated environment with M062X/6-311 + *G*(2*d*,2*p*) optimizations, followed by M062X/6-311 + *G*(3*df*,2*p*) singlepoint calculations performed on optimized structures. To represent sPEEK, this work employed the model molecule sPEEK1, one of the model molecules originally implemented in reference 7. This molecule is displayed in Fig. 2. As seen in the figure, the carbon atoms of the aromatic ring are numbered 1–6. These carbon atoms are sites for radical attachment as well as proton attachment, as seen in reference 6 and in this work.

This study found that addition of H radicals to carbon atoms at sites 1 and 4 of SPEEK1 produces adduct structures in which the C–OCH_3_ linkage, the portion of the model corresponding to the ether bridge in the sPEEK polymer, becomes extremely fragile; in fact, breaking of these bonds following addition are found to be slightly exergonic processes. Transition state optimizations found that these bond breaking reactions have low free energy barriers; only 4–8 kcal/mol relative to the adducts, and below the relative free energy of the sPEEK1 + H reactants. Similarly, the addition of H radical to the carbon at site 6 produces an adduct structure in which C–S bond breaking is slightly exergonic and in which the barrier to bond breaking is small (~ 7 kcal/mol).

The addition–elimination reactions of sPEEK1 with H suggest similar reactions might occur upon OH addition to sites 1–6 in the aromatic ring of that molecule. In the case of addition at sites 1 and 4, the expected result is phenoxy radicals such as those detected in the ex situ spin trap experiments [21]. Addition of OH radicals at site 6 might also be expected to produce HSO_3_ radicals in the same fashion as addition of H radicals. It may be speculated that such reactions may compete with the acid-catalyzed elimination reaction in the phenoxy radicals.

One of the goals of this work is the computation of the reaction pathway, including thermodynamics and barrier heights, for OH addition followed acid-catalyzed water elimination in sPEEK as proposed in reference 1. The aromatic ring of the model molecules is thus assumed as the initial attack site. This work provides the first computational density-functional determination of this mechanism.

Another goal of this work is computation of barriers and thermodynamics for addition–elimination reactions analogous to those reported for sPEEK and H radicals in reference 6. These reactions to C–O chain breaking and C–S bond breaking reactions following addition of OH at sites 1,4 and 6. Thus, the aromatic ring is assumed as the initial site of OH attack for all reaction mechanisms in this work. With the computation of the thermodynamics of and barriers to these reactions, the relative competitiveness of addition–elimination reactions with respect to acid-catalyzed water elimination reactions may be assessed.

These computations employ solvated density functional M062X/6-311 + *G*(2*d*,2*p*) calculations of the potential energy surface of the reactions of sPEEK model molecules with OH radicals and H_3_O^+^, with singlepoint calculations with a larger basis set at all optimized geometries, to provide improved energetics. The chosen methods provide complete consistency with the calculations implemented within reference 6.

In this manuscript, all species optimized with these solvated density functional M062X/6-311 + *G*(2*d*,2*p*) calculations will be named in bold. Cartesian coordinates for all these structures are provided, in the order mentioned in this article, in the Supplementary Information for this article (see Online Resource 1).

The model molecules include SPEEK1 as noted above, as well as additional molecules SPEEK1PH1 and SPEEK1PH4 also displayed in Fig. 2. The latter two replace –OCH_3_ moieties at sites 1 and 4 with phenyl rings. Model molecules SPEEK1PH1 and SPEEK1PH4 provide more realistic polymer models and hence improved thermodynamics and barriers for the computations of C–O bond breaking reactions at sites 1 and 4. The following, second section of this work describes the methods of these calculations in detail. The third section, Results, describes the formation of sPEEK hydroxyl radicals and discusses all the optimized reaction pathways in detail, displaying energetics with respect to OH-sPEEK adduct and protonated OH-sPEEK adduct molecules. The fourth section, Discussion and Conclusion, then summarizes the results in terms of reactions on the sPEEK + OH + H_3_O^+^ potential energy surface, with one of the models SPEEK1, SPEEK1PH1 and SPEEK1PH4 assuming the role of sPEEK in calculation of the energetics.

## Computational methods

The Gaussian 16 [22] electronic structure packages were used for all calculations.

As discussed above, reference 6 computed reactions of the sPEEK1 model molecule with H radicals in a solvated environment. This work calculates structures with M062X/6-311 + *G*(2*d*,2*p*) optimizations, with improved energetics provided by M062X/6-311 + *G*(3*df*,2*p*) singlepoint calculations performed on optimized structures. The recently developed M062X hybrid density functional of Zhao and Truhlar [23] was chosen because this functional was originally recommended for computation of systems where thermochemistry, kinetics, and noncovalent interactions are important [23, 24], making it suitable for the reaction thermodynamics and reaction barrier heights. It has been shown to provide good performance in calculating bond dissociation energies for reactions involving radicals [25]. It has very recently been implemented to calculate the reaction pathways, including transition states, of reactions of OOH and OH radicals in aqueous solution [26–28]

Within reference 6, additional calculations of the enthalpies of a number gas-phase reactions involving radicals also illustrated that M062X/6-311 + *G*(3*df*,2*p*) singlepoint calculations following M062X/6-311 + *G*(2*d*,2*p*) optimizations can very accurately reproduce the energetics of a number of reactions involving breaking of C–O and C–C bonds to form H radicals as well as radicals of carbon and oxygen atoms such as CH_3_, OCH_3_ and OH radicals.

Thermochemistry, kinetics, and noncovalent interactions are also important within this present work, and accuracy in the computed energetics of reactions involving OCH_3_ and OH radicals is also desirable. Therefore, the use of the functional and basis sets chosen in reference 6 continues in this work.

Unrestricted density functional M062X [23], calculations with the 6-311 + *G*(2*d*,2*p*) basis set (M062X/6-311 + *G*(2*d*,2*p*)) optimized all reactants, products, intermediates, and transition states, and determined reaction energetics. For the initial M062X/6-311 + *G*(2*d*,2*p*) calculations, frequency calculations characterized each structure as a minimum or transition state, and provided enthalpy and free energy corrections to the base energy. The connectivity of all transition states to reactants and products was determined by Intrinsic Reaction Coordinate (IRC) calculations.

Single-point M062X/6-311 + *G*(3*df*,2*p*) calculations at optimized geometries provided improved energetics. For M062X/6-311 + *G*(3*df*,2*p*)//M062X/6-311 + *G*(2*d*,2*p*) calculations, the enthalpy and free energy corrections are taken as those found by M062X/6-311 + *G*(2*d*,2*p*) frequency calculations. For all calculations, solvent (water) modeling is provided by the integral equation formalism polarized continuum model [29] (IEFPCM or PCM) as implemented in the Gaussian program. The free Avogadro [30] program was used to visualize optimized structures and also visualize computed imaginary vibrational modes in transition state structures.

The potential energy surface generated is thus compatible with recent work on the model sPEEK1 molecule with hydrogen atoms [6].

## Results

### Addition of OH to model molecule SPEEK1

The solvated M062X/6-311 + *G*(*d*,*p*) optimizations show that the addition of OH radical (OH) to sites 1–6 of model molecule SPEEK1 is thermodynamically spontaneous and occurs without reverse barrier. Table 1 shows the enthalpies and free energies of formation of adducts at each of the six sites. While enthalpies and free energies of addition of H radicals to SPEEK1 were shown to diverge by up to ~ 8.4 kcal/mol [6], the enthalpies and free energies for the formation of OH adducts are more similar, varying by only 2.4 kcal/mol in the case of the enthalpy and 1.4 kcal/mol in the case of the free energy. Table 1 also shows the enthalpy and entropy of addition provided by solvated B3LYP/6-311 + *G*(*d*) calculations as shown in reference 7. In general, the computations of this work predict more exothermic and exergonic additions than reference 7.

**Table 1:** Enthalpies (*H*_form_) and free energies (*G*_form_) of OH adduct radicals

Site on SPEEK1	OH adduct	*H*_form_	*G*_form_
1	SPEEK1OH1	− 23.2, − *23.9*	− 13.2, − *13.9*
2	SPEEK1OH2	− 18.3, − *19.1* (* − 13.7*)*	− 8.3, − *9.1* (*− 4.4)
3	SPEEK1OH3	− 19.2, − *20.2* (*− 19.0)	− 9.0, − *10.0* (*− 7.8)
4	SPEEK1OH4	− 20.0, − *20.6*	− 10.0, − *10.6*
5	SPEEK1OH5	− 21.9, − *23.0* (*− 16.0)	− 11.8, − *13.0* (*− 6.1)
6	SPEEK1OH6	− 21.4, − *21.5*	− 12.4, − *12.6*

Values are relative to SPEEK1 + OH and are in kcal/mol. M062X/6-311 + *G*(2*d*,2*p*) values are followed by M062X/6-311 + *G*(3*df*,2*p*)//M062x/6-311 + *G*(2*d*,2*p*) values, in italic. Values with asterisks (*) are solvated B3LYP/6-311 + *G*(*d*) values for the addition reaction, in aqueous solvent, from reference 7, for comparison

### Elimination following addition of OH at site 1

**Fig. 3:**
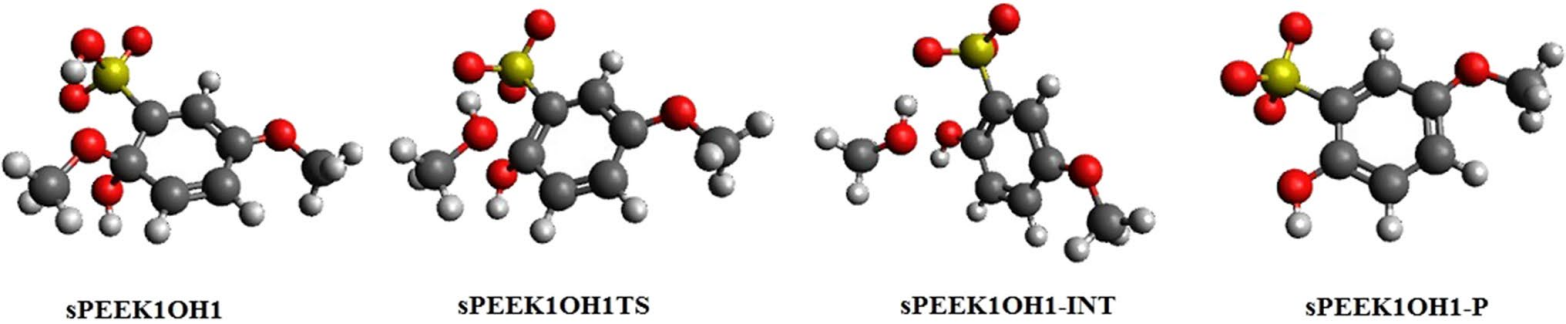
Optimized structures for elimination reaction following addition of OH to the carbon atom at site 1 of sPEEK1

The SPEEK1OH1 adduct forms at site 1 with enthalpies and free energies relative to SPEEK1 + OH as found in Table 1. A C–O bond breaking transition state SPEEK1OH1TS appears at a small enthalpy and free energy relative to the adduct, and connects the adduct to intermediate SPEEK1OH1-INT, at lower enthalpy and free energy to the adduct. This structure is a hydrogen-bonded complex of methanol to a molecule in which –OH replaces –OCH_3_ at site 1 and the H has been abstracted from the sulfonyl group at site 6, leaving an unpaired electron. The overall reaction leading to the separated products is slightly exothermic (− 3.4 kcal/mol) and exergonic (− 15.1 kcal/mol), as summarized in Table 2. Figure 3 displays optimized structures on the reaction pathway.

**Table 2:** Enthalpies (*H*_form_) and free energies of (*G*_form_) of adducts from OH and sPEEK1 and sPEEK1PH1 model molecules resulting from addition at site 1, relative to the OH and sPEEK model reactants

Reactant with OH	*H*_form_	Gf_orm_	*H*_rel_, transition state	*G*_rel_, transition state	*H*_rel_ intermediate	*G*_rel_ intermediate	*H*_rel,_ product	*G*_rel,_ product
SPEEK1	− 23.2, − *23.9*	− 13.2, − *13.9*	5.3, *4.7*	6.3, *5.6*	− 9.0, − *9.6*	− 10.5, − *11.0*	− 2.9, − *3.4*	− 15.7, − *15.1*
SPEEK1PH1	− 24.7, − *25.4*	− 14.2, − *14.9*	3.8, *3.7*	4.9, *4.7*	− 15.5, − *16.3*	− 17.0, − *17.9*	− 7.1, − *7.7*	− 20.1, − *20.7*

This is followed by enthalpies (*H*_rel_) and free energies (*G*_rel_) of optimized structures of the addition–elimination reaction relative to the sPEEK-OH adduct. M062X/6-311 + *G*(2*d*,2*p*) values are followed by M062X/6-311 + *G*(3*df*,2*p*)//M062x/6-311 + *G*(2*d*,2*p*) values, in italic. Values are in kcal/mol

The optimized structure for this radical is named SPEEK1OH1-P. The final products, methanol (CH3OH) and SPEEK1OH1-P are found without reverse barrier to the intermediate and are at an enthalpy of − 2.9 (− 3.4) relative to the adduct. Figure 3 displays optimized structures on the pathway of this reaction. The elimination/hydrogen abstraction process reported here is also shown to occur at site 1 following addition of H radicals at site 1 [6]; namely, the elimination process in that reaction also produces methanol and a species with an unpaired electron on the –SO_3_ group.

M062X/6-311 + *G*(2*d*,2*p*) calculations also computed the addition of OH to site 1 of SPEEK1PH1 to form SPEEK1PH1OH1, and determined a transition state SPEEK1PH1OH1TS for breaking of the C–O bond at site 1. As is also shown in Table 2, replacement of a methyl group for a phenyl group at site 1 of the model molecule produces a transition state with an enthalpy of only 3.7 kcal/mol relative to the reactant, which is 1.0 kcal/mol lower than in the case of the methyl substituent. The free energy of the transition state is also lowered by phenyl replacement of the methyl group, 3.7 kcal/mol as opposed to 4.7 kcal/mol. Phenyl replacement at site 1 might be anticipated to produce a similar lowering of barriers by ~ 1.0 kcal/mol to reaction for the bond-breaking reaction following addition of H radical at site 1 of SPEEK1 as described in reference 6. The barrier relative to the sPEEK-H would then be 6.3, rather than 7.3, kcal/mol [6].

IRC calculations for this transition state confirm the connectivity of SPEEK1PH1OH1TS–SPEEK1PH1OH1 in one direction of the reaction; in the other direction, IRC calculations connect the transition state to SPEEK1PH1OH1-INT. This structure is a hydrogen-bonded complex of separated products phenol (PHENOL) and SPEEK1OH1-P.

Table 2 summarizes the binding enthalpy and free energy of the OH adduct to SPEEK1 and SPEEK1PH1, as well as the relative enthalpy and free energy of the transition state and products.

### Elimination following addition of OH at site 4

Table 1 shows the enthalpy and free energy of formation of the SPEEK1OH4 adduct from SPEEK1 + OH. Energetics of structures for a C–O bond breaking reaction are shown in Table 3. A C–O bond breaking transition state SPEEK1OH4TS appears at an enthalpy of 19.3 kcal/mol and free energy of 18.9 kcal/mol relative to the adduct. This transition state lies at a much higher enthalpy or free energy relative to the adduct than is the case for bond breaking at site 1.

**Table 3:** Enthalpies (*H*_form_) and free energies of formation (*G*_form_) of adducts of OH with sPEEK1 and sPEEK1PH4 model molecules resulting from addition at site 4, relative to the OH and sPEEK model reactants

Reactant with OH	*H*_form_	Gf_orm_	*H*_rel_, transition state	*G*_rel_, transition state	*H*_rel_ intermediate	*G*_rel_ intermediate	*H*_rel,_ product	*G*_rel,_ product
SPEEK1	− 23.2, − *23.4*	− 13.1, − *13.9*	19.0, *19.3*	18.6, *18.9*	4.6, *5.0*	2.6, *3.0*	9.1, *9.7*	− 2.6, − *2.1*
SPEEK1PH4	− 21.6, − *22.3*	− 10.6, − *11.3*	9.3, *9.5*	9.3, *9.6*	− 16.5, − *16.6*	− 17.0, − *17.2*	− 10.1, − *9.6*	− 22.0, − *21.6*

This is followed by enthalpies (*H*_rel_) and free energies (*G*_rel_) of optimized structures of the addition–elimination reaction relative to the sPEEK-OH adduct. M062X/6-311 + *G*(2*d*,2*p*) values are followed by M062X/6-311 + *G*(3*df*,2*p*)//M062x/6-311 + *G*(2*d*,2*p*) values, in italic. Values are in kcal/mol

IRC calculations connect SPEEK1OH4TS–SPEEK1OH4 in one direction and to SPEEK1OH4-INT in the other, the latter is an intermediate complex in which an OCH_3_ radical is hydrogen-bonded to the sulfonyl hydrogen on the phenolic product molecule. Intermediate SPEEK1OH4-INT lies at an enthalpy of ~ 5.0 kcal/mol relative to the adduct. As indicated by the structure of the intermediate exit complex, no hydrogen abstraction from the sulfonyl group takes place during the addition–elimination reaction at site 4, and the final products include a CH_3_O radical (CH_3_O) and a phenolic molecule in which OH has replaced OCH_3_ substituent at site 4 (SPEEK1OH4-P). The final separated products SPEEK1OH4-P + methoxy (CH_3_O) are found at an enthalpy of 9.7 relative to the adduct; *G* is − 2.1 relative to the adduct, for a slightly exergonic reaction.

M062X/6-311 + *G*(2*d*,2*p*) calculations also computed the addition of OH to site 1 of SPEEK1PH4 to form SPEEK1PH4OH4, and determined a transition state SPEEK1PH4OH4TS for breaking of the C–O bond at site 4. IRC calculations were carried out to establish the connectivity of this transition state to SPEEK1PH4OH4 in one direction and to sPEEK1PH4OH4-INT, a hydrogen bonded complex of the final products, PHENOXY and SPEEK4OH4-P.

**Fig. 4:**
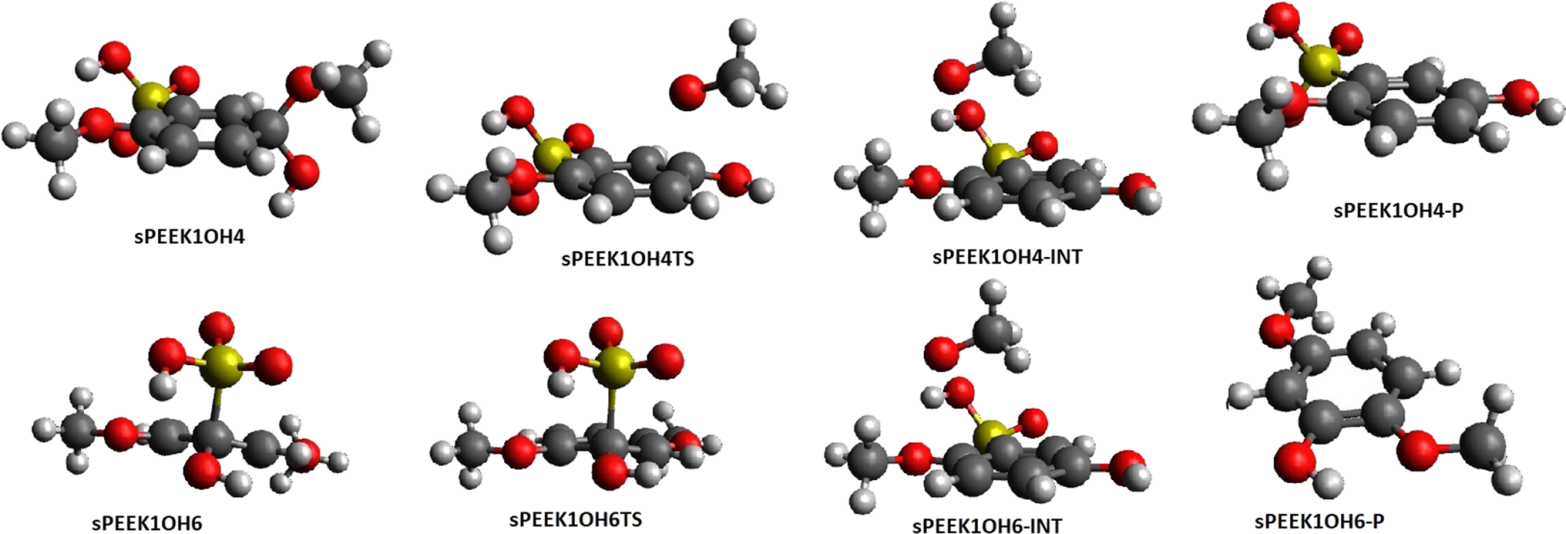
Optimized structures for reactions following OH addition to sPEEK1 at site 4 (top) and site 6(bottom)

Table 3 summarizes the binding enthalpy and free energy of the OH adduct to site 4 of SPEEK1 and SPEEK1PH4, the relative enthalpy and free energy of the transition state and products. Figure 4 displays optimized structures on the reaction pathway.

Table 3 shows that substitution of phenyl for the OCH_3_ moiety at site 4 lowers the enthalpy and free energy barriers represented by the transition state by ~ 10 kcal/mol. Phenyl replacement at site 4 might be anticipated to produce a similar lowering of barriers by ~ 10 kcal/mol to reaction for the bond-breaking reaction following addition of H radical at site 4 of SPEEK1 as described in reference 6. The free energy barrier to C–O bond breaking relative to the sPEEK-H adduct would then be lowered from 15.8 kcal/mol [6] to merely 5 kcal/mol.

### Elimination following addition of OH at site 6

#### Elimination of the HSO_3_ radical

Table 1 shows that the formation of the SPEEK1OH6 adduct from SPEEK1 + OH is exothermic and exergonic. The enthalpy and free energy of the bond breaking transition state SPEEK1OH6TS are displayed in Table 4. IRC calculations connect this transition state to SPEEK1OH6 in one direction and to a hydrogen-bonded intermediate SPEEK1OH6-INT in the other. This intermediate is as an HSO_3_ radical hydrogen bonded to the OH group on the phenolic product molecule. The separated products are the HSO_3_ radical HSO_3_ and the phenol product SPEEK1OH6-P. The C–S bond breaking reaction is slightly exothermic and occurs spontaneously. The barrier to this reaction is very small relative to SPEEK1OH6, as noted in Table 4. This result is similar to the calculated free energy of the barrier to C–S breaking relative to the adduct formed when an H radical attached at site 6; in that case, the free energy barrier was found to be only 2.4 kcal/mol [6]. Figure 4 displays optimized structures on the reaction pathway.

**Table 4:** Enthalpies (*H*_form_) and free energies of formation (*G*_form_) of adducts of OH with the sPEEK1 model molecule resulting from addition at site 6, relative to the OH and sPEEK model reactants

Reactant with OH	*H*_form_	Gf_orm_	*H*_rel_, transition state	*G*_rel_, transition state	*H*_rel_ intermediate	*G*_rel_ intermediate	*H*_rel,_ product	*G*_rel,_ product
SPEEK1	− 21.3, − *21.5*	− 12.4, − *12.6*	− 0.1^a^, *0.6*	0.6, *1.3*	− 11.3, − *10.5*	− 12.5, − *11.7*	− 3.6, − *2.6*	− 15.7, − *14.7*

This is followed by enthalpies (*H*_rel_) and free energies (*G*_rel_) of optimized structures of the addition–elimination reaction relative to the sPEEK-OH adduct. M062X/6-311 + *G*(2*d*,2*p*) values are followed by M062X/6-311 + *G*(3*df*,2*p*)//M062x/6-311 + *G*(2*d*,2*p*) values, in italic. Values are in kcal/mol

^a^The electronic energy of transition state SPEEK1OH6TS is only 0.14 kcal/mol greater than the energy of sPEEK1OH6

#### Thermodynamics of other elimination reactions following OH addition at site 6

Reference 6 suggests that in the case of OH radical addition at site 6, H_2_SO_4_ (H_2_SO_4_) elimination might occur; the other product would be the sPEEK molecule in which the C–S bond at site 6 is broken, leaving an unpaired electron at C6; this molecule is referred to as sPEEK1OH6-P2. Additional reactions following OH addition at site 6 might involve the production of HSO_4_ (HSO_4_) producing a sPEEK1 molecule in which –SO_3_H is replaced by –H, referred to as sPEEK1OH6-P3. Another possibility would be elimination of H_2_SO_3_ (H_2_SO_3_), the other product being a sPEEK1 molecule in which a phenoxy radical replaces –SO_3_H at site 6, referred to as sPEEK1OH6-P4. Table 5 summarizes the enthalpy and free energy of the reactions discussed above.

**Table 5:** Enthalpy (ΔH) and free energy (ΔG) of elimination reactions of sPEEK1OH6

Products of elimination reaction of sPEEK1OH6	ΔH	ΔG
sPEEK1OH6-P2 + H_2_SO_4_	23.3, *18.8*	11.5, *7.1*
sPEEK1OH6-P3 + HSO_4_	25.5, *21.6*	13.8, *9.9*
sPEEK1OH6-P4 + H_2_SO_3_	− 11.5, − *8.2*	− 24.7, − *21.4*

M062X/6-311 + *G*(2*d*,2*p*) values in kcal/mol are followed by M062X/6-311 + *G*(3*df*,2*p*)//M062x/6-311 + *G*(2*d*,2*p*) values in kcal/mol, in italic. Values are relative to sPEEK1OH6

### Protonation and water elimination following OH addition at site 3

#### Structure and thermodynamics of protonated sPEEK1OH3 complexes; partial charge of likely protonation sites for sPEEK-OH adducts

Site 3 of SPEEK1 is chosen for computational investigation of the water elimination oxidative process suggested for the degradation of sPEEK membranes [1, 10]. Table 1 notes that the SPEEK1OH3 moiety forms spontaneously and without barrier. The M062X/6-311 + *G*(2*d*,2*p*) geometry optimizations do not locate a minimum corresponding to addition of a proton to the hydroxyl oxygen attached to site 3. The nearest sites for protonation to the hydroxyl group are the carbons at site 2 (SPEEK1OH3H2+) and site 4 (SPEEK1OH3H4+). Protonation at these sites (corresponding to transfer of a proton from an optimized solvated hydronium, H_3_O+ ) to form the protonated moiety and an optimized solvated water molecule (H_2_O) is both exothermic and exergonic in the case of site 3, as shown in Table 6. In contrast, addition to site 4 is slightly endothermic and endergonic, as also shown in Table 6.

**Table 6:** Enthalpy (Δ*H*) and free energy (Δ*G*) of protonation reactions of SPEEK1OH3

Reaction	Δ*H*	Δ*G*
SPEEK1OH3 + H_3_O+ -> SPEEK1OH3H2+ + H_2_O	− 22.2, − *22.0*	− 22.0, − 21.9
SPEEK1OH3 + H3O+ -> SPEEK1OH3H4+ + H_2_O	0.8, *1.1*	0.8, *1.2*

M062X/6-311 + *G*(2*d*,2*p*) values are followed by M062X/6-311 + *G*(3*df*,2*p*)//M062x/6-311 + *G*(2*d*,2*p*) values, in italic. Values are in kcal/mol

The difference in the free energy of protonation may be a function of the partial Mulliken charge on the aromatic carbons adjacent to the site of attachment of the OH adduct. The optimized M062X/6-311 + *G*(2*d*,2*p*) SPEEK1OH3 structure exhibits a Mulliken charge of − 0.16453 on site number 2 and a Mulliken charge of + 0.316640 on site 5. The exergonic addition of proton at site 2 may be a function of the partial negative charge at site 2 to form SPEEK1OH3H2+ , while the partial positive charge at site 4 may correspond to the endergonicity of protonation at that site.

Table 7 summarizes the partial charges on adjacent aromatic carbons for adducts sPEEK1OH1 through sPEEK1OH6. All adducts other than sPEEK1OH2 exhibit a negative Mulliken charge on at least one aromatic carbon adjacent to the attachment site of the OH radical. An additional solvated M062X/6-311 + *G*(2*d*,2*p*) calculation of the OH adduct of benzene, BZ-OH, provides a partial negative charge of − 0.101056 on sites 2 and 6, the sites adjacent to the attachment site (referred to as site 1).

**Table 7:** Mulliken charges on carbon atoms adjacent to site of OH attachment in OH adduct molecules

OH adduct	Adjacent site with least partial charge, followed by Mulliken charge	Adjacent site with greatest partial positive charge, followed by Mulliken charge
SPEEK1OH1	C6: − 0.295895	C2: + 0.311261
SPEEK1OH2	C1: + 0.213092	C3: + 0.229474
SPEEK1OH3	C2: − 0.164503	C4: + 0.078565
SPEEK1OH4	C3: − 0.207607	C5: + 0.011635
SPEEK1OH5	C6: − 0.523895	C4: + 0.008081
SPEEK1OH6	C1: − 0.249535	C5: − 0.241307
BZ-OH	C2: − 0.101056	C6: − 0.101056

This work presents acid-catalyzed water elimination reactions occurring following protonation of sPEEK1OH3 at site 2.

#### Barrier to protonation of sPEEK1OH3

The mechanism of the acid catalyzed elimination reaction studied in this work requires that the SPEEK1OH3 hydroxylated aromatic ring is protonated by a hydronium molecule. The protonation of aromatic rings by hydronium has been the subject of theoretical investigation [31]. Earlier gas-phase ab initio calculation [31] of the benzene-hydronium potential energy surface finds that a transition state for protonation connects a benzene-hydronium encounter complex to an exit complex for protonated benzene to water. The energy change from separated reactants to separated product is found to be − 10.5 kcal/mol, and the transition state for the protonation process is found to be energetically lower than both the reactant and the product, connecting in one direction to an encounter complex of hydronium and benzene, and in the other direction to an exit complex of protonated benzene and water.

**Fig. 5:**
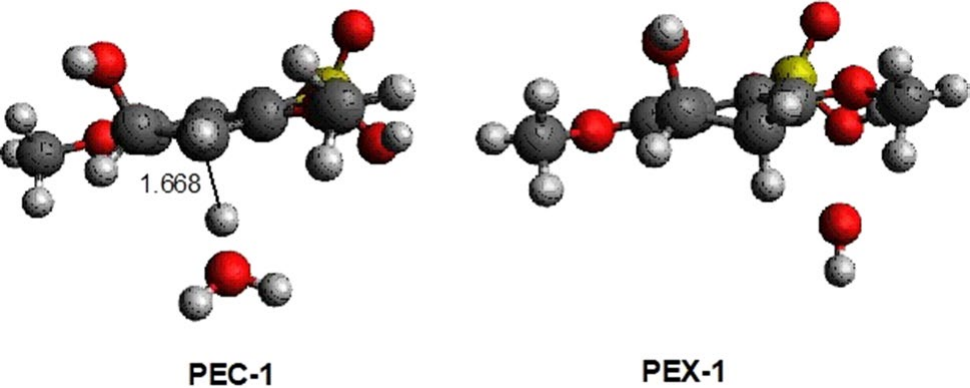
Optimized structures of hydronium-SPEEK1 encounter complex PEC-1 and sPEEK1 + -water exit complex PEX-1. The C–H separation in PEC-1 is 1.668 angstroms

**Fig. 6:**
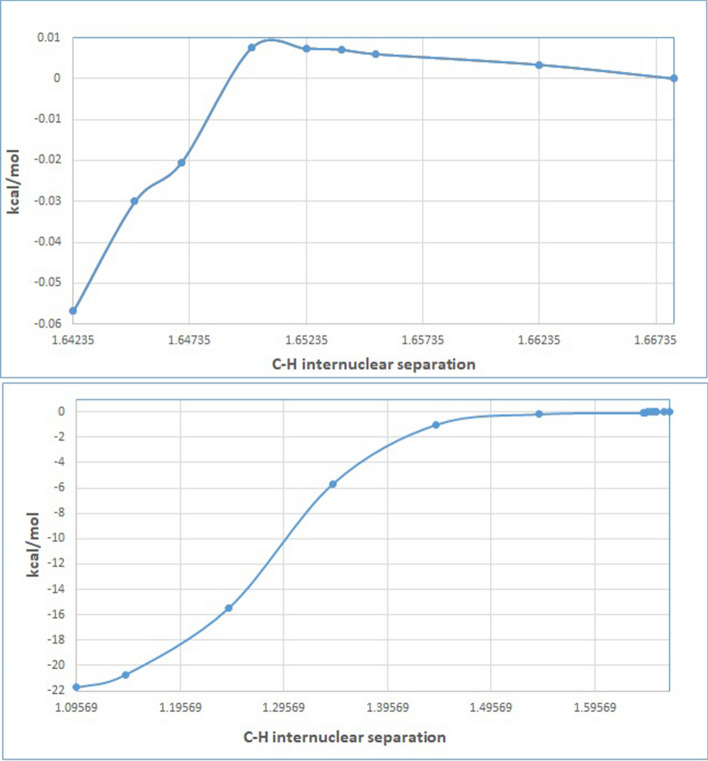
Plot of electronic energy as a function of C–H separation. The top shows the maximum at 1.65000 angstroms relative to PEC-1, the bottom plot shows the progression of energies from 1.66811 angstroms to 1.09569 angstroms (PEC-1 to PEX-1)

M062x/6-311 + *G*(2*d*,2*p*) optimizations locate an encounter complex of SPEEK1OH3 with the hydronium molecule (H_3_O+), PEC-1. (see Fig. 5). A protonation transition state from PEC-1 to a complex of water and SPEEK1OH3H2+ is not presented in this work. A series of constrained geometry optimizations [32] in which the C–H internuclear distance shown in Fig. 6 is fixed at values between 1.66811 angstroms, the optimized distance C–H distance for structure SPEEK1OH3H2+ , and values near 1 angstrom. All other nuclear coordinates are permitted to optimize. The resulting energies from these calculations are plotted in Fig. 6. Online Resource 2 of the Supplementary Information provides a table displaying the C–H internuclear distances and energies of the resulting structures relative to PEC-1. Online Resource 2 also provides Z-matrices for the structures resulting from the constrained optimizations.

Figure 6 displays an extremely flat potential energy surface. Of the points plotted, the maximum potential energy relative to PEC-1 is found at a C–H distance of 1.65000; the relative energy is only ~ 0.07 kcal/mol greater than PEC-1. At distances smaller than 1.65000, the potential energy decreases to values below the electronic energy of PEC-1. The smallest C–H distance for constrained optimization represented in this figure is 1.14235 angstroms; when this geometry is made a starting point for a full optimization, a complex of SPEEK1OH3H2+ and water is optimized. This complex, PEX-1, has a C-H internuclear separation of only 1.09569 angstroms. The electronic energy is 21.7 kcal/mol lower than that of PEC-1.

Figure 6 suggests that little or no energetic barrier exists to protonation of SPEEK1OH3.

#### Elimination of water

**Fig. 7:**
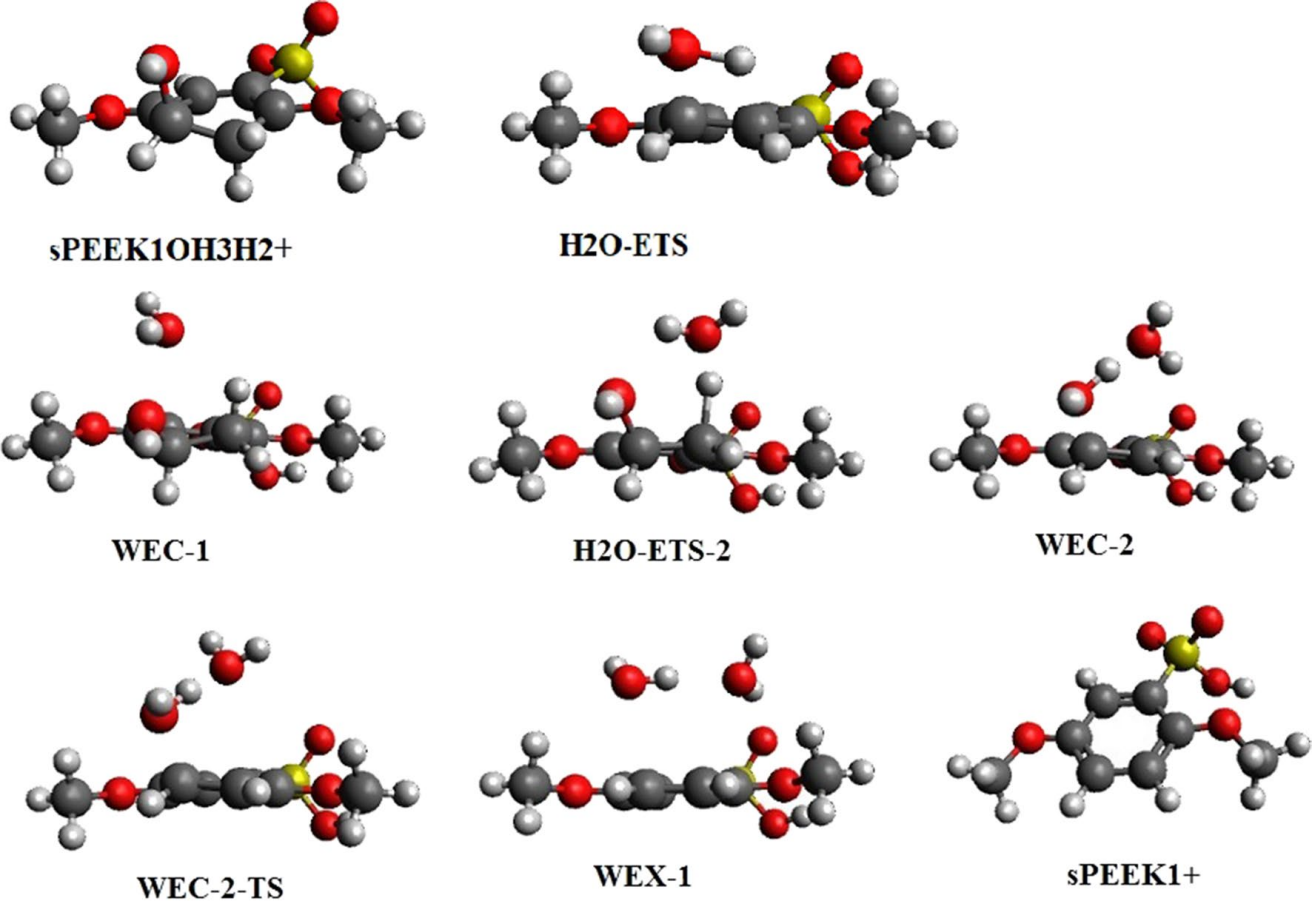
Structures of protonated sPEEK-hydroxy adduct SPEEK1OH3H2+ , and transition states, intermediates, and product for water elimination reactions

SPEEK1OH3H2+ may eliminate water to form a cationized SPEEK1 molecule (SPEEK1+). Elimination occurs through the transition state H_2_O-ETS as displayed in Fig. 7. IRC calculations establish the connectivity of this transition state to SPEEK1OH3H2+ and to the SPEEK1 cation SPEEK1+ and a water molecule in the other. The thermodynamics of the reaction pathway are displayed in Table 8. As seen in Table 8, the barrier to the reaction is extremely high relative to the protonated hydroxyl-sPEEK complex.

**Table 8:** Enthalpy (*H*_rel_) and free energy (*G*_rel_) of optimized structures on the pathway of the water elimination reaction of SPEEK1OH3H2+

Optimized structure(s)	*H*_rel_	*G*_rel_
SPEEK1OH3H2+	0, *0*	0, *0*
H_2_O-ETS	38.7, *38.6*	39.5, *39.4*
SPEEK1+ + H_2_O	− 8.7, − *8.2*	− 19.5, − *19.0*

M062X/6-311 + *G*(2*d*,2*p*) values relative to SPEEK1OH3H2+ are followed by M062X/6-311 + *G*(3*df*,2*p*)//M062x/6-311 + *G*(2*d*,2*p*) values, in italic. Values are in kcal/mol

Water elimination after protonation may also occur via a second process involving a reaction of SPEEK1OH3H2+ with an explicit water molecule; note that this process lies on the potential energy surface of the reactions of sPEEK model molecules with OH radicals and H_3_O^+^.

The resulting reaction may be written.

SPEEkK1OH3H2+  + H_2_O → SPEEK1+  + 2H_2_O.

The transition state H_2_O-ETS-2 connects to a hydrogen-bonded complex of the reactants, water and SPK1OH3H2+ , referred to as WEC-1. In the other direction, this transition state connects to minimum WEC-2, which exhibits one water molecule hydrogen bonded to a second water; the second water interacts with an SPEEK1+ molecule via a donative interaction between a lone pair on the water and the half-empty aromatic bonding orbital on the cation. This is a metastable structure with enthalpy and free energy greater than the final products; the final products can be reached by transition state WEC-2-TS, which connects WEC-2 to a hydrogen bonded exit complex of a SPEEK1 cation and two water molecules, referred to as WEX-1. The separated products may be reached from WEX-1 lie at an enthalpy of − 8.2 and free energy of − 9.1 relative to SPK1OH3H2+  + H_2_O. Figure 7 displays optimized structures on the reaction path. The energetics of this reaction relative to SPEEK1OH3H2+  + H_2_O are displayed in Table 9.

**Table 9:** Enthalpy (*H*_rel_) and free energy (*G*_rel_) of optimized structures on the pathway of the water elimination reaction of SPEEK1OH3H2+ with water co-catalyst

Optimized structure(s)	*H*_rel_	*G*_rel_
SPEEK1OH3H2+ + H_2_O	0, 0	0, 0
WEC-1	− 2.2, − *2.1*	6.1, 6.2
H_2_O-ETS-2	12.0, *12.2*	22.9, *23.1*
WEC-2	2.1, *2.1*	12.2, *12.2*
WEC-2-TS	2.0, *2.1*^b^	12.2, *12.3*
WEX-1	− 18.6, − *18.3*	− 12.0, − *11.6*
SPEEK1+ + 2H_2_O	− 8.7, − *8.2*	− 19.5, − *19.0*

M062X/6-311 + *G*(2*d*,2*p*) values relative to SPEEK1OH3H2+  + H_2_O are followed by M062X/6-311 + *G*(3*df*,2*p*)//M062x/6-311 + *G*(2*d*,2*p*) values, in italic. Values are in kcal/mol

^b^The electronic energy of WEC-2-TS is only 0.33 (0.39) kcal/mol greater than WEC-2

While hydronium catalyzes this elimination reaction by protonation, the water molecule acts as the co-catalyst for hydrogen atom transfer from a carbon atom to the OH group. The participation of water as a co-catalyze produces a relative barrier of 23.1 kcal/mol versus 39.4 kcal/mol, or a lowering of the barrier by 16.3 kcal/mol. A similar lowering of a hydrogen-transfer barrier appears in a computational study of the transfer of a hydrogen atom from sulfur to oxygen within the thioformic acid molecule in an gas-phase environment [33]; here, a barrier to transfer of 33 kcal/mol is lowered by 21 kcal/mol when a water molecule is included to facilitate the transfer.

## Discussion and conclusion

The thermodynamic formulation of transition state theory [34, 35] expresses rates of reaction in terms of the equilibrium between reactants and transition state, typically presenting rate constants which depend exponentially on the negative of the free energy difference between reactants and transition state. In this formulation, the lower the free energy of a transition state is, relative to the reactants, the faster the reaction might be expected to proceed.

**Fig. 8:**
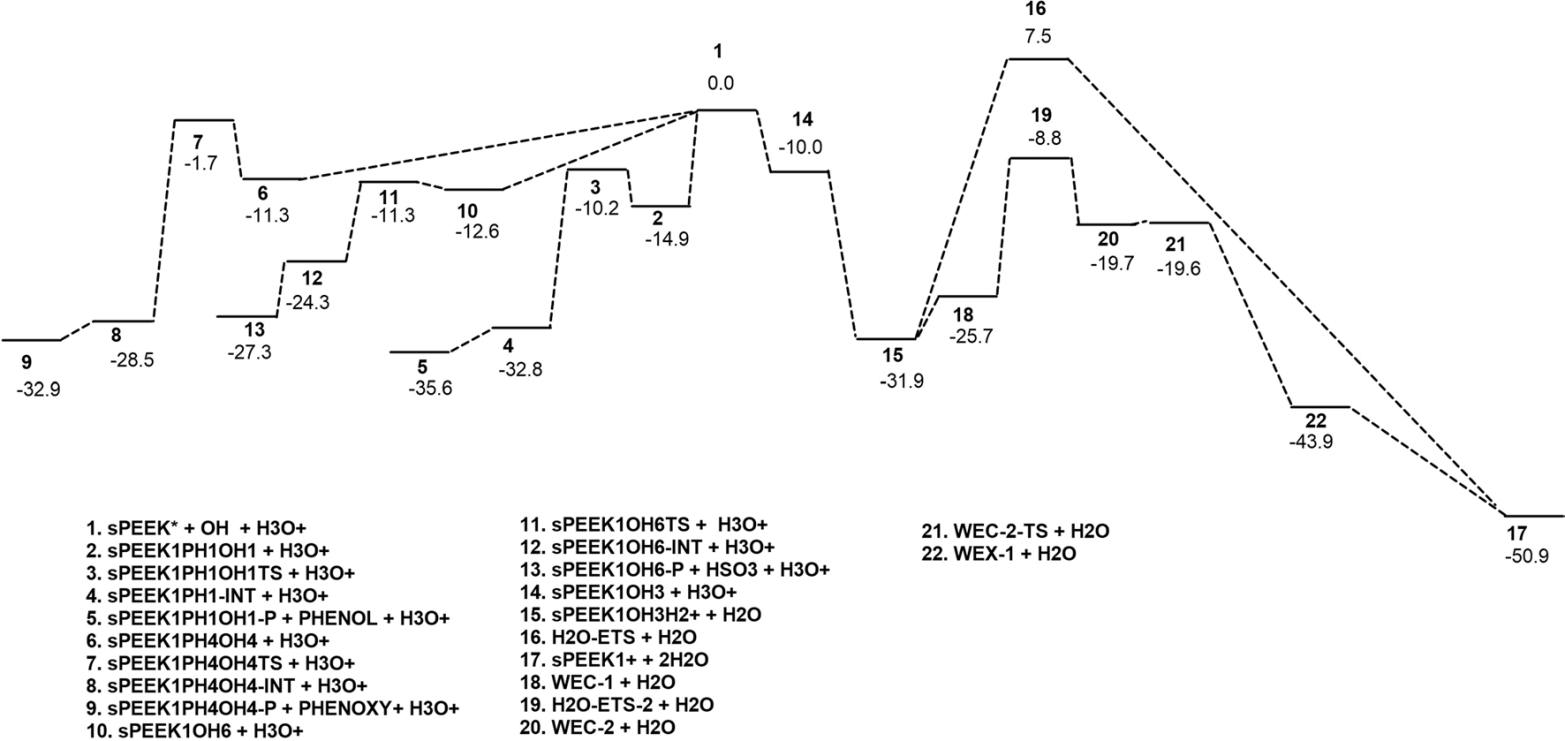
Reaction energy diagram showing M062X/6-311 + *G*(3*df*,2*p*)//M062X/6-311 + *G*(2*d*,2*p*) free energies for optimized structures in this work. Optimized structures are shown as numbers, a key to which number corresponds to which structure appears at the bottom of the diagram. All energetics shown are M062X/6-311 + *G*(3*df*,2*p*)//M062X/6-311 + *G*(2*d*,2*p*) free energies relative to the M062X/6-311 + *G*(3*df*,2*p*)//M062X/6-311 + *G*(2*d*,2*p*) free energy of reactants, which consist of OH, H_3_O+ , and sPEEK*, where sPEEK* is one of the model molecules presented in Fig. 2. This appears at the point of 1 in the diagram. Structures for the addition–elimination reaction following OH addition at site 1 appear as structures 2–5, here the identity of sPEEK* for the purpose of relative energetics is sPEEK1PH1. Structures 6–9 are optimized structures corresponding to the elimination reaction following OH addition at site 4, here the identity of sPEEK* for the purpose of relative energetics is sPEEK1PH4. Structures 10–13 are optimized structures corresponding to the elimination reaction following the addition of OH at site 6, here the identity of sPEEK* for the purpose of relative energetics is sPEEK1. Structures on the pathway for acid-catalyzed water elimination following OH addition at site 3 to form sPEEK1OH3 (14) appear as 14,15,16, and 17, the product sPEEK1+  + 2H_2_O; structures 18–22 are optimized structures for acid-catalyzed water elimination with water co-catalyst, also leading to 17, the product sPEEK1+  + 2H_2_O. The addition of a proton from H3O+ to sPEEK1OH3 to form sPEEK1OH3H2+ and H_2_O is shown as barrierless as discussed in Sect. 3.5.2

Figure 8 displays a reaction energy diagram for the reactions discussed in this work. In this diagram, M062X/6-311 + *G*(3*df*,2*p*)//M062X/6-311 + *G*(2*d*,2*p*) free energies of optimized structures are reported relative to the M062X/6-311 + *G*(3*df*,2*p*)//M062X/6-311 + *G*(2*d*,2*p*) free energy of reactants, which consist of OH, H_3_O+ , and one of the model molecules presented in Fig. 2. For reactions following addition at site 6, and for the acid-catalyzed water elimination reactions, sPEEK1 is the model molecule; in the case of the chain-breaking reactions at site 1 or 4, results are presented relative to the corresponding phenyl-substituted species sPEEK1PH1 or sPEEK1PH4, respectively.

Table 10 summarizes computed M062X/6-311 + *G*(3*df*,2*p*)//M062X/6-311 + *G*(2*d*,2*p*) free energies of products and transition states for reactions studied in this work. As in Fig. 8, all energetics in this table are reported relative to the free energy of reactants, which consist of OH, H_3_O+ , and one of the model molecules presented in Fig. 2. Again, sPEEK1 is the model molecule for reactions following addition at site 6 and for the acid-catalyzed water elimination reactions; for the chain-breaking reactions at site 1 or 4, results are presented relative to sPEEK1PH1 or sPEEK1PH4, respectively.

**Table 10:** M062X/6–311 + *G*(3*df*,2*p*)//M062X/6–311 + *G*(2*d*,2*p*) free energy (*G*_rel_) of transition states and products relative to sPEEK model molecule + H_3_O^+^  + OH, in kcal/mol

sPEEK model	Transition state	G_rel_	Product	*G*_rel_
sPEEK1PH1	SPEEK1PH1OH1TS	− 10.1	PHENOL + SPEEK1PH1OH1-P	− 35.6
sPEEK1PH4	SPEEK1PH4OH4TS	− 1.7	SPEEK1PH4OH4-P + PHENOXY	− 32.9
sPEEK1	SPEEK1OH6TS	− 11.3	SPEEK1OH6-P + HSO3	− 27.2
sPEEK1	H_2_O-ETS	7.5	sPEEK1 + + 2H_2_O	− 50.9
sPEEK1	H_2_O-ETS-2	− 8.8	sPEEK1+ + 2H_2_O	− 50.9
sPEEK1	N/A	N/A	sPEEK1OH6-P2 + H_2_SO_4_	− 5.5
sPEEK1	N/A	N/A	sPEEK1OH6-P3 + HSO_4_	− 2.7
sPEEK1	N/A	N/A	sPEEK1OH6-P4 + H_2_SO_3_	− 33.9

This work presents the first density functional computation of the acid-catalyzed water elimination reaction of hydroxyl radicals with the sPEEK polymer. This reaction, like all others computed in this work, proceeds from the addition of an OH radical to a carbon on the aromatic ring of the polymer. Figure 8 and Table 10 show that the reaction is the most exergonic of the reactions studied by at least 14.4 kcal/mol. However, direct elimination of water after protonation at an aromatic carbon adjacent to the hydroxy addition site must proceed through a transition state with a free energy greater than that of the reactants by 7.5 kcal/mol. The participation of a water molecule acting as a co-catalyst to effect the transfer of the H atom from the adjacent carbon creates a much lower barrier to the reaction; the free energy of the transition state with the water co-catalyst is 8.8 kcal/mol lower than the sPEEK1 + OH + H_3_O^+^ reactants.

The main chain of the sPEEK polymer contains ether-bridged aromatic rings; some of which contain sulfonyl groups for the purpose of proton exchange. Chain breaking elimination reactions would be predicted to result from the addition of OH radicals to aromatic carbons bonded to the oxygen atoms of the ether bridge. In the event that no -SO_3_H groups are adjacent to these carbons, the best model for the addition–elimination reaction is provided by the computed reaction pathway for the reaction of OH with sPEEK1PH4. While the chain breaking reaction is exergonic, the free energy of the transition state for this reaction is only slightly lower than that of the reactants at − 1.7 kcal/mol. If an adjacent –SO_3_H group is present, a chain-breaking reaction may occur with transfer of an H atom from –SO_3_H to the oxygen atom; the best model for that process in this work is the computed reaction of OH with sPEEK1PH1; the free energy relative to reactants of the transition state for this reaction, is substantially lower, − 10.1 kcal/mol. Addition elimination reactions producing –SO_3_H are modeled by the addition of OH to sPEEK1 at site 6; this also has a low relative free energy, − 11.3 kcal/mol.

The acid-catalyzed water elimination reaction, co-catalyzed by water, has a transition state with a low free energy relative to reactants. Calculations of protonated adducts of SPEEK1OH3 have produced results suggesting that the exergonicity of protonation of sPEEK-OH adducts adjacent to the site of OH attachment may correspond to negative partial charges on the adjacent carbon atoms. The distribution of partial charges in OH adducts displayed in Table 7 would then suggest that many aromatic carbon sites on the sPEEK polymer, including those with no sulfonyl groups in the aromatic ring (as modeled by BZ-OH), are likely sites for OH addition followed by protonation at an adjacent carbon atoms with a negative partial charge. The reaction hence might be expected to be a large fraction of the degradation reactions of the sPEEK polymer with OH. Direct addition–elimination chain breaking reactions may only happen at sites where aromatic carbons attach to bridging ether oxygens, and the free energy of the barrier to reaction is greater than that of reactants; this might be expected to be an extremely small fraction of such degradation reactions.

Reactions such as the one reported for sPEEK1 and sPEEK1PH1, in which a hydrogen atom is transferred from a sulfur to an oxygen, or addition elimination reactions producing –SO_3_H, following addition at site 6, have very low free energy barriers, lower than that of the acid-catalyzed, water co-catalyzed elimination reaction, and these reactions might be expected to compete somewhat with a predominant acid-catalyzed, water co-catalyzed elimination reaction.

Additional computational study might further elucidate mechanisms for other degradation reactions. The absence of observation of HSO_3_ product in the experiments described in references [10, 11] is puzzling in light of the extremely low free energy for the barrier to formation of this product following addition of OH radicals to site 6 reported in this work and in the case of H radicals as discussed in reference 6. One possible explanation may be the presence of a competing reaction that produces oxo-acids of sulfur; these closed-shell sulfur compounds would be undetectable by EPR or ESR methods [10, 11] implemented in the experiments described.

In addition for the reaction producing HSO_3_ after addition at site 6, Table 10 includes the possible elimination reactions at site 6 as presented in Table 5. Transition states for these reactions are not reported in this work. H_2_SO_3_ production, however, is highly exergonic with respect to sPEEK1OH6. The free energy barrier must be at least equal to the relative free energy of sPEEK1OH6, which, as seen in Table 5, is − 12.6 kcal/mol relative to the sPEEK + OH + H_3_O^+^ reactants. The products are H_2_SO_3_, which as a closed-shell molecule would not be detected by electron resonance experiments, and a phenoxy radical; phenoxy radicals are observed in the experiments in degradation involving sPEEK discussed in reference 11. Future work will investigate the transition state to H_2_SO_3_ elimination reactions following addition at site 6.

As seen in Table 5, the reactions producing HSO_4_ and H_2_SO_4_ are endergonic with respect to sPEEK1OH6, meaning that free energy barriers to these reactions relative to the reactants must be at least as high as the free energies of the products. Therefore, reactions that produce closed-shell H_2_SO_4_ or open-shell HSO_4_ might be considered uncompetitive as these free energies of products are higher than the free energy barriers reported in Table 10.

While uncompetitive, more computational information on possible reactions producing H_2_SO_4_ may assist in providing more complete information on possible degradation reactions of sPEEK and OH radicals. H_2_SO_4_ might be produced in a reaction arising from attack of the S atom by the OH radical. In the case of Nafion, computations showed that OH attack on the S atom of the HSO_3_ moiety led to a reaction producing H_2_SO_4_ [5]. This reaction was found to have a ΔE of − 23 kcal/mol/, the barrier to the reaction was found to be 18.7 kcal/mol relative to the reactants. In the case of sPEEK, the analogous reaction producing H_2_SO_4_ and the radical product sPEEK1OH6-P2 is shown in Table 10 to be only mildly exergonic relative to the sPEEK + OH + H3O + reactants. The free energy of any barrier to this possible minor product, as noted above, is necessarily greater than the free energy of the products, but its relative energy with respect to the reactants remains unknown.

The solvated B3LYP calculations discussed in reference 7 find endergonic H-abstraction reactions for the reaction of OH with sPEEK1. Specifically, free energies of H abstraction from site 2 of sPEEK by OH to form water and sPEEK1 radicals are found to have values of approximately − 25 or − 39 kcal/mol, with the more exergonic value occurring in the case of deprotonated sPEEK. H abstraction from the –SO_3_H group by OH radicals might also occur, possibly with a low barrier to reaction; Reference 5 notes that in the case of Nafion this abstraction reaction is found to be barrierless and exothermic (ΔE = − 5.3 kcal/mol). Future work will confirm the exergonicity of these reactions in neutral and anionic species, as well as determining transition states and barrier heights for H-abstraction reactions.

## Supplementary Information

Below is the link to the electronic supplementary material.

Supplementary file1 (DOCX 35 KB)

Supplementary file2 (DOCX 28 KB)
