# Supplementary material for: Density functional theory investigation of mechanisms of degradation reactions of sulfonated PEEK membranes with OH radicals in fuel cells: addition–elimination reactions and acid catalyzed water elimination
Source: Theor Chem Acc. 2023 Apr 25;142(5):49. doi: 10.1007/s00214-023-02981-2 (PMC10129967; doi:10.1007/s00214-023-02981-2)
Supplement: Supplementary file 1 — Supplementary file1 (DOCX 35 KB) [file 214_2023_2981_MOESM1_ESM.docx]

Online Resource 1:

Article Title: Density functional theory investigation of mechanisms of degradation reactions of sulfonated PEEK membranes with OH radicals in fuel cells: Addition-elimination reactions and acid catalyzed water elimination

Journal: Theoretical Chemistry Accounts

Authors: Jonathan E. Stevens^a,b^, Courtney M. Pefley^b^, Alice Piatkowski^c^, Zachary R. Smith^d^, Nikolina Ognanovich^e^

^a^corresponding author. email: [stevenje@udmercy.edu](mailto:stevenje@udmercy.edu) phone:313-993-1048

^b^Department of Chemistry and Biochemistry, University of Detroit Mercy, Detroit MI 48221

^c^University of Detroit Mercy, School of Dentistry, Detroit MI 48208

Affiliation: Department of Chemistry and Biochemistry, University of Detroit Mercy, Detroit, MI 48221

Content-

Cartesian Coordinates for all optimized structures (angstroms), in order mentioned in the article.

**SPEEK1**

C -0.22248 -0.26344 0.01475

C 0.95472 -0.97961 0.00133

C 2.17258 -0.30084 0.00020

C 2.17384 1.08810 0.00628

C 0.97592 1.80190 0.00864

C -0.23771 1.13677 0.01493

O 3.28355 -1.07736 -0.00893

O -1.45765 1.73489 0.00131

C -1.49258 3.15981 0.00562

C 4.53937 -0.41501 -0.01673

H -1.00434 3.55699 -0.88372

H -1.01232 3.54783 0.90313

H -2.54248 3.43025 0.00285

H 4.66095 0.19838 0.87709

H 5.29079 -1.19725 -0.02673

H 4.64559 0.20624 -0.90706

S -1.74818 -1.14298 0.08748

O -1.52622 -2.51424 -0.26663

O -2.42267 -0.82586 1.31501

O -2.57532 -0.53004 -1.11954

H -2.71317 0.41825 -0.94369

H 1.01163 2.87993 0.00361

H 3.09983 1.64172 0.00386

H 0.93883 -2.05974 -0.00874

**SPEEK1PH1**

C 0.98899 -0.61657 -0.04718

C 2.30707 -0.49713 0.33207

C 2.96460 0.71625 0.12617

C 2.27992 1.76562 -0.47608

C 0.95207 1.61852 -0.87653

C 0.28335 0.43106 -0.64837

O 4.25083 0.76826 0.53227

O -0.99239 0.16095 -1.09393

C 4.95835 1.98178 0.31268

H 4.49145 2.80546 0.85311

H 5.95979 1.81623 0.69383

H 5.00390 2.21580 -0.75116

S 0.16569 -2.15406 0.22543

O 1.12315 -3.14425 0.61926

O -1.00627 -1.92958 1.02263

O -0.28243 -2.57340 -1.23934

H -0.93791 -1.93016 -1.55967

H 0.48221 2.47900 -1.33241

H 2.76549 2.71138 -0.65775

H 2.83000 -1.32945 0.77976

H -1.28980 2.58969 0.04475

C -2.15471 1.95519 0.10618

C -2.11278 0.68102 -0.43025

C -3.22279 -0.14815 -0.40327

H -3.16882 -1.13864 -0.83120

C -4.39243 0.30754 0.18841

H -5.25941 -0.33728 0.21364

C -4.44842 1.57788 0.75174

H -5.35911 1.92608 1.21696

C -3.32836 2.39574 0.70809

H -3.36118 3.38839 1.13432

**SPEEK1PH4**

C -1.50562 -0.24324 -0.13861

C -0.29506 -0.85475 -0.41560

C 0.80081 -0.06358 -0.71238

C 0.68071 1.31815 -0.73159

C -0.53497 1.92642 -0.45366

C -1.64763 1.14728 -0.15312

O 1.97841 -0.68706 -1.05556

O -2.88200 1.63592 0.10960

C -3.05460 3.05260 0.10009

H -2.82966 3.45748 -0.88540

H -2.41916 3.51575 0.85316

H -4.09733 3.22331 0.34117

S -2.88608 -1.24939 0.29258

O -2.61394 -2.60641 -0.07902

O -3.29906 -0.94637 1.63364

O -4.01602 -0.76220 -0.70915

H -4.21994 0.16945 -0.51278

H -0.60766 3.00188 -0.48357

H 1.54365 1.92452 -0.96981

H -0.20825 -1.93127 -0.40592

H 4.29190 0.67710 2.62954

C 4.30607 0.33338 1.60471

C 3.10153 0.07767 0.95950

H 2.16221 0.21695 1.47502

C 3.12333 -0.36465 -0.35709

C 4.32539 -0.55840 -1.02524

H 4.30801 -0.90527 -2.04864

C 5.51936 -0.30410 -0.36667

H 6.45497 -0.45376 -0.88701

C 5.51598 0.14584 0.94941

H 6.44741 0.34720 1.45844

**OH**

O 0.00000 0.00000 0.10806

H 0.00000 0.00000 -0.86448

**sPEEK1OH1**

C -0.17064 -0.37722 0.12209

C 1.00691 -1.03607 0.03899

C 2.22854 -0.32706 -0.02868

C 2.21137 1.09144 -0.12070

C 1.04684 1.77231 -0.04568

C -0.27580 1.12100 0.24297

O 3.33224 -1.07733 -0.05642

O -1.27113 1.51999 -0.69756

C -1.75774 2.85376 -0.57091

C 4.59839 -0.42383 -0.13472

H -0.95857 3.58184 -0.70750

H -2.23395 2.99929 0.39612

H -2.49017 2.98273 -1.36142

H 4.72986 0.25361 0.70772

H 5.33963 -1.21348 -0.09286

H 4.68892 0.11972 -1.07413

S -1.65835 -1.30001 0.06921

O -1.35912 -2.70320 0.00423

O -2.57509 -0.81915 1.06532

O -2.24432 -0.93732 -1.36413

H -2.25782 0.03855 -1.42596

H 1.04372 2.84966 -0.14273

H 3.13077 1.63495 -0.27702

H 1.02923 -2.11633 -0.00034

O -0.69825 1.43697 1.55120

H -0.28978 2.26389 1.82933

**SPEEK1OH2**

C -0.34459 -0.41575 0.00636

C 0.77849 -1.23440 0.03737

C 2.07302 -0.63816 -0.08448

C 2.22357 0.70130 -0.19541

C 1.07171 1.64911 -0.10718

C -0.26010 0.95374 -0.11869

O 3.08021 -1.54496 -0.09261

O -1.39767 1.65569 -0.18369

C -1.35266 3.04520 -0.54663

C 4.40050 -1.03680 -0.20240

H -0.99044 3.14983 -1.56737

H -0.72392 3.59601 0.14599

H -2.37845 3.38944 -0.48372

H 4.62995 -0.37475 0.63370

H 5.05850 -1.89875 -0.18077

H 4.52983 -0.49800 -1.14212

S -1.93434 -1.16494 0.15813

O -1.80872 -2.57938 -0.03630

O -2.59422 -0.66008 1.32953

O -2.71308 -0.64104 -1.12312

H -2.85405 0.31663 -1.03104

H 3.19447 1.15923 -0.30281

H 0.68584 -2.30521 0.12629

O 1.16455 2.48150 1.06236

H 1.29454 1.90329 1.82213

H 1.11484 2.36207 -0.93348

**SPEEK1OH3**

C -0.46900 -0.34120 -0.01824

C 0.63909 -1.16889 -0.05668

C 1.90450 -0.62785 -0.12825

C 2.14492 0.85181 -0.06117

C 0.88658 1.65458 -0.18719

C -0.33274 1.09306 -0.12177

O 2.92862 -1.47766 -0.18179

O -1.51687 1.75789 -0.18729

C -1.45975 3.17713 -0.28135

C 4.24247 -0.99063 -0.47819

H -0.95171 3.47733 -1.19757

H -0.94354 3.59170 0.58409

H -2.48884 3.51793 -0.29858

H 4.55295 -0.25143 0.25478

H 4.88786 -1.86047 -0.43507

H 4.26622 -0.56903 -1.48203

S -2.05307 -1.04810 0.17547

O -1.97202 -2.46562 -0.03293

O -2.67267 -0.54395 1.37110

O -2.86730 -0.49345 -1.07351

H -2.91020 0.47637 -0.99658

H 0.52190 -2.24266 -0.03826

O 2.79891 1.08503 1.19959

H 3.14490 1.98320 1.19595

H 1.01039 2.72120 -0.29603

H 2.83335 1.13945 -0.86242

**SPEEK1OH4**

C -0.39604 -0.25370 0.05954

C 0.75036 -0.95474 0.14207

C 2.08614 -0.28252 0.26017

C 1.97335 1.21368 0.25580

C 0.79129 1.87446 0.15609

C -0.42748 1.17239 0.04725

O 2.85406 -0.79267 -0.79923

O -1.63653 1.74018 -0.08590

C -1.72256 3.16743 -0.09153

C 4.14986 -0.22034 -0.95229

H -1.16144 3.57625 -0.92991

H -1.35035 3.56890 0.84903

H -2.77610 3.39587 -0.20218

H 4.66361 -0.15324 0.00577

H 4.70007 -0.88618 -1.61022

H 4.08909 0.76636 -1.41212

S -1.92687 -1.13840 0.03062

O -1.67194 -2.52171 -0.23860

O -2.70703 -0.76091 1.17433

O -2.63653 -0.58654 -1.27698

H -2.86286 0.34922 -1.13848

H 0.78651 2.95391 0.16683

H 2.89362 1.76887 0.36985

H 0.75060 -2.03516 0.14384

O 2.73496 -0.72248 1.45201

H 2.37204 -0.23731 2.19999

**SPEEK1OH5**

C 0.32650 -0.15280 0.03636

C -0.93912 -0.94524 -0.00541

C -2.14709 -0.05838 0.11539

C -2.07565 1.30798 0.02319

C -0.84044 1.95688 -0.08845

C 0.36567 1.21008 -0.06233

O -3.25892 -0.76926 0.27195

O 1.58418 1.79761 -0.10469

C 1.66288 3.22325 -0.16557

C -4.50077 -0.07032 0.33593

H 1.19356 3.67136 0.70837

H 1.19872 3.59122 -1.07875

H 2.72200 3.45442 -0.17416

H -4.67314 0.47697 -0.59000

H -5.26260 -0.82991 0.46675

H -4.50526 0.61401 1.18342

S 1.80538 -1.07240 0.08846

O 1.52265 -2.37848 0.61615

O 2.52814 -0.96064 -1.15165

O 2.67430 -0.36097 1.22026

H 2.85084 0.55275 0.93808

H -0.81537 3.03040 -0.17139

H -2.97237 1.90762 0.04379

O -1.02759 -1.74166 -1.19275

H -0.95496 -1.15353 -1.95283

H -0.96150 -1.67837 0.80254

**SPEEK1OH6**

C 0.32849 -0.13757 0.74093

C -1.02507 -0.71311 0.79836

C -2.08779 -0.10791 0.20255

C -1.94449 1.16070 -0.42509

C -0.71325 1.82240 -0.38812

C 0.38463 1.25158 0.20013

O -3.26985 -0.76658 0.25427

O 1.58696 1.81092 0.33737

C 1.77154 3.12066 -0.19336

C -4.42072 -0.13327 -0.29053

H 1.09408 3.82435 0.28940

H 1.60389 3.11874 -1.27002

H 2.79990 3.38604 0.02304

H -4.30588 0.03185 -1.36180

H -5.24325 -0.81881 -0.11702

H -4.62175 0.81271 0.21236

S 1.29517 -1.26585 -0.46236

O 1.22735 -2.60087 0.08123

O 0.92615 -1.03952 -1.83353

O 2.80064 -0.74485 -0.34993

H 3.06314 -0.73988 0.58431

H -0.64294 2.81053 -0.81545

H -2.78222 1.64139 -0.90188

H -1.14004 -1.68407 1.26026

O 1.09150 -0.24597 1.91252

H 0.79033 -1.01510 2.40990

**SPEEK1OH1TS**

C -0.18560 -0.42711 0.19929

C 0.97592 -1.06203 -0.05611

C 2.21816 -0.37499 -0.00155

C 2.24504 1.00343 0.31607

C 1.09171 1.67579 0.55721

C -0.22055 1.02491 0.47705

O 3.29289 -1.11323 -0.25545

O -0.90432 1.57393 -0.95506

C -1.44044 2.90879 -0.91765

C 4.57859 -0.49024 -0.21622

H -0.62899 3.58955 -0.67778

H -2.23807 2.97655 -0.18276

H -1.81888 3.12732 -1.91146

H 4.77682 -0.09655 0.77931

H 5.29000 -1.27205 -0.45381

H 4.63582 0.30493 -0.95770

S -1.74571 -1.26930 -0.00201

O -1.45097 -2.51952 -0.67534

O -2.37241 -1.40206 1.29856

O -2.48132 -0.30389 -0.88226

H -1.67050 0.85696 -1.05554

H 1.11325 2.72054 0.83772

H 3.18419 1.52947 0.39203

H 0.97350 -2.11197 -0.31503

O -1.12943 1.37821 1.42058

H -0.89829 2.22520 1.82027

**SPEEK1OH1-INT**

C -0.16751 -0.50039 0.27410

C 0.97067 -1.00531 -0.25595

C 2.22157 -0.35701 -0.03531

C 2.29025 0.82898 0.74348

C 1.14699 1.33441 1.27837

C -0.09949 0.68940 1.05971

O 3.25181 -0.92635 -0.59666

O -0.87394 1.96898 -1.38229

C -1.55677 3.17166 -1.07256

C 4.56415 -0.35192 -0.45656

H -0.81917 3.97013 -1.03093

H -2.05900 3.10852 -0.10420

H -2.29873 3.42087 -1.83405

H 4.85283 -0.34927 0.59164

H 5.21803 -0.99761 -1.02844

H 4.56933 0.65444 -0.86753

S -1.75247 -1.28865 -0.06357

O -1.41268 -2.43662 -0.88908

O -2.29891 -1.61860 1.24280

O -2.50844 -0.25283 -0.77524

H -1.50523 1.23734 -1.31073

H 1.16937 2.23390 1.87844

H 3.23275 1.32489 0.91207

H 0.94674 -1.89856 -0.86348

O -1.21065 1.15257 1.57222

H -1.06412 1.95768 2.08868

**SPEEK1OH1-P**

H 0.51631 -1.87531 0.00000

C 0.66507 -0.80513 0.00000

C -0.39528 0.03721 0.00000

C -0.15882 1.44420 0.00000

C 1.16812 1.95295 0.00000

H 1.31602 3.02433 0.00000

C 2.23215 1.10664 0.00000

H 3.23681 1.49786 0.00000

C 1.99682 -0.29528 0.00000

O 2.93932 -1.19517 0.00000

C 4.32339 -0.79949 0.00000

S -2.07750 -0.61779 0.00000

O -2.66796 -0.09171 1.22360

O -1.88701 -2.06235 0.00000

O -2.66796 -0.09171 -1.22360

H 4.88233 -1.72626 0.00000

H 4.53986 -0.22499 -0.89731

H 4.53986 -0.22499 0.89731

O -1.19700 2.24078 0.00000

H -0.93989 3.17370 0.00000

**CH3OH**

C 0.04709 0.66502 0.00000

O 0.04709 -0.75591 0.00000

H -0.44134 1.06663 0.88902

H 1.08639 0.98353 0.00000

H -0.44134 1.06663 -0.88902

H -0.86292 -1.05964 0.00000

**SPEEK1PH1OH1**

C -1.23894 0.60750 0.13739

C -2.45910 0.04739 -0.02985

C -2.63123 -1.35367 0.04355

C -1.49577 -2.19301 0.21174

C -0.26522 -1.66089 0.37183

C -0.00846 -0.18719 0.47346

O -3.88369 -1.79132 -0.09862

O 0.98987 0.23281 -0.49254

C -4.12231 -3.19806 -0.06093

H -3.80083 -3.61300 0.89300

H -5.19341 -3.31713 -0.17363

H -3.60592 -3.69355 -0.88152

S -1.06312 2.33249 -0.11277

O -2.35288 2.92416 -0.33003

O -0.18316 2.88953 0.87551

O -0.33879 2.41472 -1.52812

H 0.47186 1.87501 -1.47568

H 0.59739 -2.30338 0.49340

H -1.60861 -3.26625 0.20007

H -3.31991 0.66542 -0.24383

H 2.79969 1.25043 1.10811

C 3.14653 0.39095 0.55316

C 2.27744 -0.25332 -0.31723

C 2.69084 -1.34685 -1.06572

H 1.99412 -1.81591 -1.74654

C 3.99371 -1.80888 -0.93106

H 4.32208 -2.65870 -1.51250

C 4.87131 -1.18002 -0.05525

H 5.88449 -1.54167 0.04738

C 4.44720 -0.08080 0.68213

H 5.13013 0.41581 1.35685

O 0.40885 0.14688 1.76760

H 0.98130 -0.55015 2.10955

**SPEEK1PH1OH1TS**

C 1.30110 -0.64700 0.23988

C 2.41080 0.01322 -0.15995

C 2.52046 1.41568 0.02585

C 1.45902 2.13304 0.62627

C 0.32680 1.48621 1.00717

C 0.14349 0.06491 0.77210

O 3.65647 1.95814 -0.38962

O -0.86612 0.00837 -0.72728

C 3.83909 3.36962 -0.24709

H 3.81822 3.64750 0.80499

H 4.81442 3.58088 -0.66810

H 3.06908 3.90577 -0.79868

S 1.11502 -2.36857 -0.13271

O 2.19398 -2.75459 -1.00591

O 0.94387 -3.13142 1.07639

O -0.21895 -2.33785 -0.92197

H -0.61461 -1.33540 -0.91710

H -0.47807 2.02100 1.49371

H 1.54787 3.19318 0.80493

H 3.22544 -0.51644 -0.63335

H -2.79039 -1.60087 0.06239

C -3.09769 -0.57389 -0.08198

C -2.15932 0.36811 -0.50572

C -2.53592 1.69838 -0.69267

H -1.79303 2.41067 -1.02476

C -3.85041 2.07860 -0.46598

H -4.14224 3.10861 -0.61634

C -4.79178 1.14110 -0.05127

H -5.81532 1.44138 0.12185

C -4.41226 -0.18250 0.13730

H -5.14006 -0.91476 0.45778

O -0.60553 -0.63714 1.64092

H -1.29180 -0.08142 2.03237

**SPEEK1PH1OH1-INT**

C -1.30201 0.66397 0.29224

C -2.39972 0.03341 -0.18594

C -2.57790 -1.36565 0.02707

C -1.59840 -2.11890 0.72824

C -0.49639 -1.48399 1.21085

C -0.32974 -0.08719 1.01892

O -3.67104 -1.87211 -0.47182

O 1.10323 -0.10092 -1.46850

C -3.94824 -3.27763 -0.33088

H -4.02373 -3.53157 0.72333

H -4.89801 -3.43002 -0.82655

H -3.16699 -3.85488 -0.81865

S -1.02926 2.41060 -0.06029

O -2.18388 2.80379 -0.85046

O -0.92607 3.05626 1.23703

O 0.23059 2.41377 -0.81852

H 0.90401 0.84873 -1.35033

H 0.26391 -2.03051 1.75208

H -1.72256 -3.17905 0.88006

H -3.14542 0.57422 -0.75032

H 2.89040 1.60614 -0.50251

C 3.15788 0.56216 -0.40366

C 2.28562 -0.41526 -0.88138

C 2.60595 -1.76384 -0.74555

H 1.91344 -2.50725 -1.11638

C 3.80037 -2.12962 -0.13975

H 4.04391 -3.17828 -0.03834

C 4.67759 -1.16183 0.33799

H 5.60485 -1.45129 0.81083

C 4.34806 0.18219 0.20292

H 5.01980 0.94449 0.57295

O 0.70944 0.55320 1.48845

H 1.34795 -0.03925 1.91266

**PHENOL**

H -2.67940 0.76841 0.00000

O -2.29488 -0.11335 0.00000

H -0.83296 2.11614 0.00000

C -0.26688 1.19341 0.00000

C -0.93414 -0.02699 0.00000

C -0.21668 -1.21879 0.00000

H -0.75420 -2.15670 0.00000

C 1.17015 -1.18317 0.00000

H 1.72347 -2.11214 0.00000

C 1.84842 0.03143 0.00000

H 2.92852 0.05367 0.00000

C 1.12221 1.21575 0.00000

H 1.63522 2.16751 0.00000

**SPEEK1OH4TS**

C -1.61003 -0.26350 0.26666

C -0.54522 -0.98199 0.70609

C 0.65827 -0.29092 1.08418

C 0.58175 1.13104 1.23901

C -0.50192 1.83543 0.78914

C -1.60758 1.15590 0.25759

O 1.45774 -0.39807 -0.63033

O -2.69373 1.74449 -0.25196

C -2.75570 3.17453 -0.26191

H -1.93488 3.58081 -0.84955

H -2.72415 3.55674 0.75622

H -3.70416 3.42120 -0.72336

S -3.07352 -1.12549 -0.22008

O -2.76442 -2.51541 -0.36836

O -4.16376 -0.71269 0.61517

O -3.32807 -0.59558 -1.69228

H -3.60351 0.33578 -1.65019

H -0.50554 2.91181 0.85489

H 1.43316 1.63549 1.67253

H -0.57303 -2.06237 0.72116

H 5.65645 -1.98283 -0.17323

C 5.00590 -1.12944 -0.30809

C 3.63264 -1.31319 -0.36154

H 3.20023 -2.30198 -0.28165

C 2.77233 -0.21834 -0.54899

C 3.33203 1.06656 -0.66041

H 2.66756 1.90822 -0.80489

C 4.70506 1.24158 -0.60254

H 5.12364 2.23437 -0.69724

C 5.54873 0.14665 -0.42551

H 6.61938 0.28795 -0.38174

O 1.57065 -0.89748 1.87064

H 1.59642 -1.84523 1.69316

**SPEEK1OH4-INT**

C -0.04599 -0.50427 -0.39232

C -1.12139 -1.34794 -0.19280

C -2.41047 -0.84317 -0.32088

C -2.59138 0.49831 -0.63330

C -1.50331 1.34180 -0.82426

C -0.21004 0.84789 -0.71136

O 0.13449 1.72378 1.91493

O 0.91936 1.58323 -0.85194

C 0.76742 2.99491 -0.98100

C -0.53309 0.74792 2.61160

H 0.22415 3.39395 -0.12509

H 0.24981 3.23920 -1.90758

H 1.77308 3.39912 -1.00602

H 0.04426 -0.17163 2.72811

H -0.87631 1.13908 3.57564

H -1.45281 0.52828 2.04275

S 1.58456 -1.14036 -0.18058

O 1.51517 -2.45174 0.39713

O 2.34799 -0.94397 -1.37991

O 2.16129 -0.21286 0.96898

H 1.99018 0.72255 0.74674

H -1.67692 2.38110 -1.05413

H -3.59284 0.89771 -0.72447

H -0.96317 -2.38311 0.07206

O -3.44089 -1.70643 -0.12133

H -4.28208 -1.24915 -0.21572

**CH3O**

H 1.05584 -0.86330 0.00000

C -0.01082 -0.57874 0.00000

O -0.01082 0.79141 0.00000

H -0.45219 -0.99779 0.90752

H -0.45219 -0.99779 -0.90752

**SPEEK1OH4-P**

C 0.09438 -0.30674 0.01671

C 1.17296 -1.17066 -0.00084

C 2.45949 -0.64665 -0.00613

C 2.63681 0.72962 -0.00090

C 1.54640 1.59187 0.00641

C 0.25610 1.08159 0.01813

O -0.87675 1.83144 0.01182

C -0.72865 3.24938 0.00267

H -0.19821 3.57219 -0.89253

H -0.19780 3.58037 0.89438

H -1.73510 3.65214 0.00167

S -1.53092 -0.98450 0.08721

O -1.48489 -2.37071 -0.27489

O -2.15963 -0.59248 1.31767

O -2.27421 -0.26402 -1.11515

H -2.28827 0.69348 -0.93475

H 1.71864 2.65645 0.00118

H 3.63743 1.14156 -0.00816

H 1.01788 -2.23963 -0.01177

O 3.49324 -1.53088 -0.01898

H 4.33310 -1.06190 -0.02536

**SPEEK1PH4OH4**

C -1.62423 -0.27689 0.10413

C -0.56159 -1.09240 0.23979

C 0.78201 -0.57245 0.65281

C 0.77358 0.89902 0.92406

C -0.32099 1.68003 0.75040

C -1.55005 1.12881 0.32770

O 1.64411 -0.92491 -0.44547

O -2.67233 1.82937 0.10172

C -2.65289 3.24022 0.33480

H -1.92544 3.71988 -0.31709

H -2.42293 3.44374 1.37875

H -3.65122 3.58906 0.09919

S -3.18766 -0.99360 -0.31032

O -2.98760 -2.33538 -0.76884

O -4.11901 -0.72685 0.74804

O -3.62460 -0.17585 -1.59707

H -3.80019 0.74520 -1.33762

H -0.25153 2.73818 0.95164

H 1.69962 1.32577 1.28130

H -0.64640 -2.15367 0.04998

H 5.99919 -1.12769 0.82902

C 5.22654 -0.60904 0.27886

C 3.92542 -1.09707 0.30001

H 3.67401 -1.99022 0.85267

C 2.93675 -0.42925 -0.41027

C 3.23704 0.71010 -1.14540

H 2.44810 1.20402 -1.69561

C 4.54021 1.19021 -1.16267

H 4.77632 2.07616 -1.73528

C 5.53638 0.53424 -0.44825

H 6.55003 0.90879 -0.46320

O 1.23763 -1.19896 1.82528

H 1.23829 -2.15406 1.68633

**SPEEK1PH4OH4TS**

C -1.61003 -0.26350 0.26666

C -0.54522 -0.98199 0.70609

C 0.65827 -0.29092 1.08418

C 0.58175 1.13104 1.23901

C -0.50192 1.83543 0.78914

C -1.60758 1.15590 0.25759

O 1.45774 -0.39807 -0.63033

O -2.69373 1.74449 -0.25196

C -2.75570 3.17453 -0.26191

H -1.93488 3.58081 -0.84955

H -2.72415 3.55674 0.75622

H -3.70416 3.42120 -0.72336

S -3.07352 -1.12549 -0.22008

O -2.76442 -2.51541 -0.36836

O -4.16376 -0.71269 0.61517

O -3.32807 -0.59558 -1.69228

H -3.60351 0.33578 -1.65019

H -0.50554 2.91181 0.85489

H 1.43316 1.63549 1.67253

H -0.57303 -2.06237 0.72116

H 5.65645 -1.98283 -0.17323

C 5.00590 -1.12944 -0.30809

C 3.63264 -1.31319 -0.36154

H 3.20023 -2.30198 -0.28165

C 2.77233 -0.21834 -0.54899

C 3.33203 1.06656 -0.66041

H 2.66756 1.90822 -0.80489

C 4.70506 1.24158 -0.60254

H 5.12364 2.23437 -0.69724

C 5.54873 0.14665 -0.42551

H 6.61938 0.28795 -0.38174

O 1.57065 -0.89748 1.87064

H 1.59642 -1.84523 1.69316

**SPEEK1PH4OH4-INT**

C -0.63329 0.66787 0.62660

C 0.42483 0.99527 1.45808

C 1.36017 1.92573 1.02467

C 1.21129 2.51663 -0.22329

C 0.16141 2.16673 -1.05927

C -0.77445 1.22530 -0.64559

O -0.15602 -1.56748 -2.16406

O -1.81616 0.78275 -1.39265

C -1.84591 1.16527 -2.76618

H -0.92372 0.85966 -3.25868

H -1.99362 2.24021 -2.85859

H -2.68946 0.64067 -3.20065

S -1.87434 -0.43203 1.22794

O -1.39475 -1.09154 2.40906

O -3.13239 0.25967 1.27638

O -1.94678 -1.56164 0.12330

H -1.98034 -1.15836 -0.76649

H 0.08692 2.62290 -2.03402

H 1.94281 3.24280 -0.54839

H 0.50857 0.52348 2.42767

H 2.04387 -3.02121 1.45612

C 1.98251 -2.35886 0.60437

C 0.92587 -2.44086 -0.25882

H 0.12863 -3.15591 -0.11951

C 0.83047 -1.54627 -1.39582

C 1.91430 -0.60715 -1.59712

H 1.85148 0.04632 -2.45625

C 2.95585 -0.54254 -0.71430

H 3.74988 0.17682 -0.85776

C 2.99653 -1.40687 0.39406

H 3.82152 -1.34371 1.08912

O 2.43890 2.28665 1.77154

H 2.44308 1.81005 2.60733

**PHENOXY**

O         -2.28889        0.00001       0.00000

H         -0.85036        2.16101        0.00000

C         -0.28814        1.23764        0.00000

C         -1.03976        0.00000      0.00000

C         -0.28814       -1.23764       0.00000

H         -0.85036       -2.16101       0.00000

C          1.07908       -1.22313        0.00000

H          1.63568       -2.14944        0.00000

C          1.77248        0.00000        0.00000

H          2.85290        0.00000       0.00000

C          1.07908        1.22313        0.00000

H          1.63568        2.14944        0.00000

**SPEEK1OH6TS**

C 0.28895 -0.02213 0.91404

C -1.05434 -0.54004 1.01833

C -2.06429 -0.00394 0.26808

C -1.83452 1.15449 -0.51583

C -0.59409 1.79133 -0.48519

C 0.44595 1.27801 0.25540

O -3.26692 -0.61716 0.34211

O 1.65592 1.81607 0.40510

C 1.93082 3.02321 -0.30250

C -4.35698 -0.05053 -0.37484

H 1.26058 3.81635 0.02689

H 1.82497 2.86382 -1.37526

H 2.95628 3.27998 -0.06341

H -4.16343 -0.05771 -1.44734

H -5.21258 -0.68021 -0.15608

H -4.55785 0.96693 -0.03907

S 1.17522 -1.33480 -0.46809

O 1.20219 -2.63046 0.18626

O 0.65037 -1.25453 -1.81263

O 2.69929 -0.81360 -0.61332

H 3.08482 -0.73270 0.27261

H -0.46754 2.71119 -1.03419

H -2.62341 1.58298 -1.11180

H -1.22870 -1.42165 1.61948

O 1.16119 -0.21527 1.95729

H 0.93812 -1.03506 2.41411

**SPEEK1OH6-INT**

C -0.64595 -0.55481 0.91205

C -1.87295 -1.09754 0.61451

C -2.83657 -0.32226 -0.03966

C -2.54869 0.98761 -0.38478

C -1.29541 1.52628 -0.07437

C -0.33950 0.77107 0.57474

O -4.01653 -0.94900 -0.29050

O 0.90928 1.19057 0.92557

C 1.28276 2.49775 0.51383

C -5.01673 -0.19697 -0.95754

H 0.64151 3.24804 0.97656

H 1.23178 2.58852 -0.57262

H 2.30650 2.63707 0.84667

H -4.67253 0.12211 -1.94222

H -5.86930 -0.85874 -1.06677

H -5.30418 0.67640 -0.37063

S 2.91361 -0.25307 -0.63537

O 1.76287 -0.64927 -1.42450

O 4.25529 -0.42567 -1.15321

O 2.88836 -1.00989 0.75698

H 1.94972 -1.04212 1.08500

H -1.08622 2.54787 -0.35170

H -3.27113 1.60704 -0.89124

H -2.10446 -2.12118 0.87692

O 0.34217 -1.26267 1.55189

H 0.06725 -2.17533 1.69330

**HSO3**

S 0.12752 0.04978 0.25577

O -1.26858 -0.63586 -0.13605

O 1.13322 -0.88813 -0.18421

H -1.99427 -0.00937 0.01608

O 0.12961 1.42560 -0.19330

**SPEEK1OH6-P**

C -0.65734 1.14412 0.00000

C 0.71113 1.28868 0.00000

C 1.53258 0.15657 0.00000

C 0.97066 -1.10987 0.00000

C -0.42304 -1.24710 0.00000

C -1.23854 -0.13523 0.00000

O 2.87157 0.40805 0.00000

O -2.60423 -0.13256 0.00000

C -3.24745 -1.39660 0.00000

C 3.73835 -0.71308 0.00000

H -2.98023 -1.96557 0.89161

H -2.98023 -1.96557 -0.89161

H -4.31341 -1.19576 0.00000

H 3.58541 -1.32443 -0.89048

H 4.74794 -0.31532 0.00000

H 3.58541 -1.32443 0.89048

H -0.84942 -2.23877 0.00000

H 1.58149 -1.99819 0.00000

H 1.15010 2.27617 0.00000

O -1.45177 2.24617 0.00000

H -2.37170 1.95391 0.00000

**H2SO4**

S -0.02569 0.14326 0.00000

O -1.30540 0.75953 0.00000

O -0.02569 -0.83643 1.23310

O 1.18793 0.89533 0.00000

O -0.02569 -0.83643 -1.23310

H 0.88088 -1.07412 1.47953

H 0.88088 -1.07412 -1.47953

**SPEEK1OH6-P2**

C 0.65819 1.46473 0.00000

C -0.69942 1.51539 0.00000

C -1.39222 0.29218 0.00000

C -0.67207 -0.89365 0.00000

C 0.72765 -0.88227 0.00000

C 1.43112 0.31642 0.00000

O -2.74945 0.37790 0.00000

O 2.78547 0.44398 0.00000

C 3.53377 -0.76118 0.00000

C -3.47247 -0.84207 0.00000

H 3.31860 -1.35300 -0.89114

H 3.31860 -1.35300 0.89114

H 4.57852 -0.46870 0.00000

H -3.24556 -1.42920 0.89130

H -4.52315 -0.57127 -0.00000

H -3.24556 -1.42920 -0.89130

H 1.25096 -1.82719 0.00000

H -1.17649 -1.84695 0.00000

H -1.25196 2.44578 0.00000

**HSO4**

S -0.01817 0.09385 0.00000

O -0.94598 -0.22123 -1.10471

O -0.94598 -0.22123 1.10471

O 0.61385 1.36611 0.00162

O 1.06949 -1.02817 0.00000

H 1.95857 -0.64115 0.00000

**SPEEK1OH6-P3**

C 0.68895 1.47462 0.00000

C -0.68895 1.47462 0.00000

C -1.39695 0.26914 0.00000

C -0.69927 -0.92808 0.00000

C 0.69927 -0.92808 0.00000

C 1.39695 0.26914 0.00000

O -2.75692 0.37419 0.00000

O 2.75692 0.37419 0.00000

C 3.49461 -0.83510 0.00000

C -3.49461 -0.83510 0.00000

H 3.27578 -1.42651 0.89082

H 3.27578 -1.42651 0.89082

H 4.54239 -0.55261 0.00000

H -3.27589 -1.42650 0.89077

H -4.54239 -0.55261 0.00000

H -3.27589 -1.42650 -0.89077

H 1.21615 -1.87516 0.00000

H -1.21615 -1.87516 0.00000

H -1.24180 2.40377 0.00000

H 1.24180 2.40377 0.00000

**H2SO3**

S 0.29485 0.34258 0.00000

O 0.29485 -0.69550 1.24130

O -1.02761 0.96092 0.00000

O 0.29485 -0.69550 -1.24130

H -0.60709 -1.02031 -1.39584

H -0.60709 -1.02031 1.39584

**SPEEK1OH6-P4**

C 0.68829 1.26798 0.00000

C -0.74458 1.32982 0.00000

C -1.50835 0.19765 0.00000

C -0.88811 -1.08554 0.00000

C 0.48890 -1.20959 0.00000

C 1.29374 -0.07652 0.00000

O -2.85152 0.35508 0.00000

O 2.61778 -0.08216 0.00000

C 3.28672 -1.34230 0.00000

C -3.67265 -0.80666 0.00000

H 3.02670 -1.90918 -0.89305

H 3.02670 -1.90918 0.89305

H 4.34625 -1.11459 0.00000

H -3.49910 -1.40714 0.89324

H -4.69519 -0.44528 0.00000

H -3.49910 -1.40714 -0.89324

H 0.92301 -2.19687 0.00000

H -1.48780 -1.98183 0.00000

H -1.21056 2.30462 0.00000

O 1.40933 2.27932 0.00000

**SPEEK1OH3H2+**

C -0.45915 -0.32507 -0.08730

C 0.65571 -1.14597 -0.22350

C 1.93779 -0.60965 -0.23382

C 2.15437 0.86643 -0.01423

C 0.95426 1.68996 -0.48007

C -0.35985 1.06320 -0.17410

O -1.45188 1.75116 -0.07580

C -1.44883 3.18980 -0.23691

C 4.29359 -0.97645 -0.25464

H -0.98940 3.45159 -1.18518

H -0.92501 3.64254 0.59942

H -2.49383 3.47001 -0.22750

S -2.04035 -1.08273 0.18307

O -1.87979 -2.48722 -0.02178

O -2.59839 -0.56382 1.39453

O -2.90224 -0.56565 -1.03716

H -3.31308 0.28576 -0.81904

H 0.53497 -2.21886 -0.26913

O 2.37211 0.97613 1.37883

H 2.83396 1.80082 1.56398

H 0.99235 1.81611 -1.56837

H 3.02541 1.19825 -0.57722

O 2.92758 -1.44232 -0.32034

H 4.89162 -1.87583 -0.18851

H 4.42659 -0.35797 0.62789

H 4.53006 -0.43095 -1.16404

H 1.02555 2.68243 -0.04023

**SPEEK1OH3H4+**

C -0.44208 -0.29106 0.04016

C 0.70778 -0.97969 0.06817

C 2.03191 -0.29797 0.21885

C 2.00015 1.09135 -0.44049

C 0.78508 1.83809 -0.06143

C -0.43702 1.15545 0.08632

O 3.06921 -1.00667 -0.38479

O -1.57927 1.72988 0.24033

C -1.69769 3.17563 0.25454

C 3.64662 -2.00103 0.45641

H -1.33249 3.57181 -0.68805

H -1.14292 3.56764 1.10178

H -2.75669 3.36234 0.36876

H 2.90517 -2.74569 0.75035

H 4.42831 -2.48125 -0.12229

H 4.07312 -1.53943 1.34800

S -1.99201 -1.16443 0.01790

O -1.70333 -2.52008 -0.31947

O -2.72549 -0.82184 1.19668

O -2.71138 -0.53233 -1.23711

H -3.28295 0.20245 -0.96303

H 0.68945 -2.06077 0.00068

O 3.12992 1.84416 -0.13584

H 3.89889 1.40091 -0.51099

H 0.84795 2.91072 0.04952

H 1.91941 0.90725 -1.52760

H 2.21913 -0.15485 1.29291

**H3O+**

O 0.00000 0.00000 0.08579

H 0.00000 0.92407 -0.22876

H 0.80026 -0.46203 -0.22876

H -0.80026 -0.46203 -0.22876

**H2O**

O 0.00000 0.00000 0.11741

H 0.00000 0.75989 -0.46962

H 0.00000 -0.75989 -0.46962

**BZ-OH**

H -0.18103 -0.79139 2.18512

C -0.19401 -0.25175 1.24740

C -0.19401 1.10518 1.22397

H -0.18187 1.65775 2.15390

C -0.20146 1.81641 0.00000

H -0.19656 2.89588 0.00000

C -0.19401 1.10518 -1.22397

H -0.18187 1.65775 -2.15390

C -0.19401 -0.25175 -1.24740

H -0.18103 -0.79139 -2.18512

C -0.19079 -1.07876 0.00000

H -1.06681 -1.73758 0.00000

O 0.90965 -2.00840 0.00000

H 1.72178 -1.49089 0.00000

**PEC-1**

C 0.54930 -0.46539 -0.06692

C -0.52954 -1.30207 0.13803

C -1.82459 -0.82254 0.03223

C -2.11063 0.58481 -0.41309

C -0.90323 1.47791 -0.26963

C 0.36095 0.94237 -0.29155

C 1.40639 3.03102 -0.74231

C -4.17066 -1.25281 0.19036

S 2.16818 -1.13767 -0.05229

O 1.48947 1.63808 -0.41793

O 2.10113 -2.47386 0.45891

O 2.81125 -0.87543 -1.30788

O 2.91351 -0.30032 1.07334

O -2.52797 0.47168 -1.77770

O -2.80167 -1.68359 0.24138

O -0.89692 1.84905 2.44680

H 0.94291 3.58339 0.07290

H 0.84206 3.16340 -1.66277

H 2.43005 3.35827 -0.87696

H 3.08685 0.59497 0.73893

H -0.37376 -2.34467 0.37486

H -2.94899 1.29774 -2.03803

H -0.82386 1.71536 1.37959

H -2.93752 0.98956 0.17443

H -4.75727 -2.15773 0.29131

H -4.37866 -0.76757 -0.75849

H -4.37696 -0.58430 1.02341

H -1.05262 2.51284 -0.54580

H -1.64152 2.43493 2.66547

H -0.08016 2.24182 2.79958

**PEX-1**

C 0.33879 -0.45409 -0.10862

C -0.79520 -1.18327 0.23112

C -2.04889 -0.58461 0.23784

C -2.21740 0.84947 -0.19710

C -0.95214 1.66736 0.05399

C 0.30362 0.93465 -0.25297

O -3.06608 -1.34033 0.51899

O 1.40294 1.53220 -0.57210

C 1.49181 2.97568 -0.54510

C -4.41082 -0.81713 0.46343

H 1.17473 3.32936 0.43132

H 0.88693 3.39032 -1.34598

H 2.53935 3.19103 -0.71078

H -4.57489 -0.32904 -0.49257

H -5.05162 -1.68282 0.56853

H -4.56353 -0.13139 1.29223

S 1.87569 -1.31299 -0.29740

O 1.69357 -2.63598 0.20907

O 2.38994 -1.08061 -1.61255

O 2.81939 -0.57661 0.74613

H 3.25807 0.17545 0.31698

H -0.71597 -2.23830 0.45099

O -2.53490 0.75603 -1.57292

H -2.95240 1.57589 -1.85762

H -1.01835 2.59306 -0.51305

H -3.02887 1.31103 0.36414

H -0.88166 1.93442 1.11430

O 1.26745 1.47523 2.30821

H 1.81853 0.70235 2.46416

H 1.06147 1.81874 3.18172

**SPEEK1+**

C -0.23508 -0.27567 0.02262

C 0.90882 -0.99521 0.01104

C 2.16549 -0.31436 0.00252

C 2.21094 1.10497 0.00596

C 1.05162 1.82000 0.01157

C -0.20153 1.15296 0.01677

O 3.20988 -1.08580 -0.01002

O -1.35354 1.75589 0.00124

C -1.42923 3.19894 -0.00302

C 4.53760 -0.52202 -0.02481

H -0.95402 3.58202 -0.90170

H -0.95646 3.58610 0.89508

H -2.48779 3.42205 -0.00454

H 4.68694 0.07709 0.86936

H 5.20377 -1.37432 -0.02902

H 4.66845 0.07152 -0.92570

S -1.79392 -1.12693 0.09642

O -1.53209 -2.51364 -0.11608

O -2.50847 -0.66595 1.24620

O -2.52392 -0.61706 -1.20730

H -2.98677 0.21731 -1.02885

H 1.08584 2.89786 0.00727

H 3.15762 1.62169 -0.00053

H 0.89853 -2.07555 0.00772

**H2O-ETS**

C -0.46822 -0.34287 -0.04851

C 0.63068 -1.16949 -0.10746

C 1.91780 -0.65231 -0.20713

C 2.15148 0.81783 -0.14208

C 0.92001 1.67098 -0.24212

C -0.34051 1.07404 -0.17885

O -1.46758 1.74168 -0.22406

C -1.42988 3.17514 -0.34056

C 4.26725 -1.05334 -0.36995

H -1.00135 3.45391 -1.30008

H -0.85024 3.59005 0.48069

H -2.46239 3.49388 -0.27985

S -2.06153 -1.05695 0.19748

O -1.93142 -2.47586 0.07732

O -2.66347 -0.46714 1.35625

O -2.87355 -0.60055 -1.08354

H -3.16107 0.32053 -0.97792

H 0.50577 -2.24233 -0.07046

O 2.47742 1.15559 1.30117

H 3.26740 1.71743 1.37503

H 1.03183 2.64449 -0.69541

H 2.97240 1.15414 -0.76456

O 2.90485 -1.50960 -0.30856

H 4.86761 -1.95399 -0.37772

H 4.50125 -0.45731 0.50837

H 4.42964 -0.48973 -1.28528

H 1.42182 1.80716 1.09878

**H2O-ETS-2**

C -0.64560 -0.45761 -0.06783

C 0.39978 -1.35649 0.03721

C 1.70772 -0.97477 -0.20984

C 2.08078 0.44039 -0.55985

C 0.89425 1.38643 -0.52916

C -0.40539 0.89971 -0.43930

O -1.48862 1.63126 -0.61381

C -1.35480 3.00663 -1.00307

C 3.99036 -1.70278 -0.48989

H -0.85423 3.07272 -1.96609

H -0.80353 3.55469 -0.24244

H -2.36726 3.38309 -1.07622

S -2.27709 -0.99026 0.29859

O -2.26021 -2.41438 0.44246

O -2.83422 -0.15913 1.32644

O -3.07402 -0.71913 -1.04720

H -3.26395 0.22956 -1.12366

H 0.20308 -2.38720 0.29514

O 1.30199 2.06949 1.96972

H 2.24991 1.84145 1.93962

H 1.05140 2.31891 -1.05522

H 2.50901 0.44133 -1.56645

O 2.61324 -1.92673 -0.14540

H 4.39989 -2.69193 -0.65614

H 4.49681 -1.20820 0.33180

H 4.07087 -1.11276 -1.39916

H 0.96497 1.83015 0.84822

H 1.21990 3.01297 2.17320

O 3.09226 0.85899 0.36614

H 3.75076 1.38731 -0.09644

**WEC-1**

C -0.69981 -0.32859 -0.09335

C 0.39552 -1.18747 -0.08997

C 1.69086 -0.71893 -0.29708

C 1.93983 0.72110 -0.67094

C 0.80557 1.65366 -0.22105

C -0.55039 1.04797 -0.19068

O -1.62665 1.77598 -0.17748

C -1.55000 3.21911 -0.21313

C 3.98395 -1.38258 -0.69917

H -1.19041 3.53762 -1.18736

H -0.90298 3.57316 0.58360

H -2.56675 3.55419 -0.05547

S -2.31771 -1.02048 0.12242

O -2.20391 -2.43697 -0.02827

O -2.92314 -0.44545 1.28554

O -3.09477 -0.51410 -1.15929

H -3.44070 0.37959 -1.00618

H 0.25046 -2.24913 0.04559

O 2.00940 0.32248 2.26590

H 2.86218 0.69821 2.02318

H 0.81550 2.54573 -0.84231

H 1.99273 0.72602 -1.76631

O 2.62216 -1.61710 -0.27359

H 4.37373 -2.37282 -0.90002

H 4.53125 -0.90028 0.10202

H 3.99852 -0.77525 -1.59812

H 1.02340 1.96223 0.80763

H 2.18806 -0.28287 2.99020

O 3.15054 1.16665 -0.11109

H 3.58617 1.77356 -0.71681

**WEC-2**

C -0.56436 -0.39619 -0.21679

C 0.51280 -1.24353 -0.25734

C 1.78948 -0.73470 -0.45759

C 2.02462 0.73389 -0.49374

C 0.81160 1.55505 -0.64536

C -0.41441 1.02049 -0.46257

O -1.57756 1.69256 -0.54007

C -1.51268 3.09974 -0.77219

C 4.11656 -1.15629 -0.84152

H -1.05870 3.30034 -1.74145

H -0.94240 3.58242 0.01998

H -2.53827 3.44892 -0.76109

S -2.14668 -1.04763 0.17003

O -2.08676 -2.47783 0.11932

O -2.65114 -0.38870 1.34100

O -3.03769 -0.62587 -1.07275

H -3.12023 0.34343 -1.08204

H 0.38366 -2.30950 -0.13918

O 0.99471 0.64828 2.55708

H 1.97586 0.91550 1.66698

H 0.94827 2.59649 -0.88685

H 2.81059 1.01696 -1.18779

O 2.78770 -1.60087 -0.53384

H 4.72792 -2.04993 -0.81783

H 4.46555 -0.44680 -0.09621

H 4.14258 -0.71948 -1.83765

H 0.51603 1.43028 2.85753

H 1.29703 0.18122 3.34543

O 2.70698 1.03552 0.87225

H 3.06731 1.93674 0.87855

**WEC-2-TS**

C -0.54928 -0.39897 -0.22465

C 0.52116 -1.24422 -0.25103

C 1.80275 -0.72948 -0.46779

C 2.01626 0.72254 -0.53532

O 2.72460 1.02432 0.97684

C 0.83276 1.54418 -0.68001

C -0.39987 1.01343 -0.48336

O -1.55577 1.68706 -0.55897

C -1.49182 3.09428 -0.80212

C 4.13255 -1.14863 -0.81329

H -1.04588 3.28590 -1.77659

H -0.91519 3.58092 -0.01739

H -2.51743 3.44253 -0.78505

S -2.13849 -1.04867 0.16328

O -2.07327 -2.47811 0.12812

O -2.64377 -0.37395 1.32339

O -3.01735 -0.64107 -1.09055

H -3.12545 0.32546 -1.09956

H 0.39624 -2.30809 -0.11224

O 0.83894 0.67554 2.56901

H 1.96952 0.93310 1.68758

H 0.96941 2.58301 -0.93090

H 2.86405 1.03450 -1.13254

O 2.79625 -1.59362 -0.53228

H 4.74252 -2.04251 -0.77716

H 4.46101 -0.43852 -0.05954

H 4.17517 -0.71135 -1.80843

H 0.37957 1.46841 2.86727

H 1.05733 0.17168 3.36091

H 3.06086 1.93349 0.99223

**WEX-1**

C -0.35829 -0.44494 -0.38923

C 0.79837 -1.14354 -0.35006

C 2.03358 -0.45552 -0.55246

C 2.03834 0.93720 -0.83504

O 2.13412 0.30776 2.12029

C 0.86536 1.62506 -0.88603

C -0.36406 0.95756 -0.65425

O -1.53143 1.53417 -0.66892

C -1.62643 2.97046 -0.76714

C 4.40129 -0.57677 -0.49421

H -1.25360 3.29566 -1.73447

H -1.06533 3.41376 0.05142

H -2.68199 3.18927 -0.67333

S -1.86990 -1.25488 0.05963

O -1.63291 -2.66106 0.07294

O -2.39667 -0.58035 1.21352

O -2.81714 -0.98206 -1.16892

H -3.21403 -0.09828 -1.10043

H 0.81702 -2.20103 -0.12943

O -0.47355 1.46858 2.11142

H 1.24015 0.67409 2.21573

H 0.87049 2.68251 -1.09726

H 2.96976 1.45525 -0.99877

O 3.09801 -1.18919 -0.44667

H 5.09996 -1.38498 -0.32296

H 4.46933 0.16753 0.29548

H 4.56338 -0.13430 -1.47371

H -0.63537 2.12298 2.79693

H -1.23425 0.87372 2.12879

H 2.20035 -0.37809 2.78916
