# Supplementary material for: Density functional theory investigation of mechanisms of degradation reactions of sulfonated PEEK membranes with OH radicals in fuel cells: addition–elimination reactions and acid catalyzed water elimination
Source: Theor Chem Acc. 2023 Apr 25;142(5):49. doi: 10.1007/s00214-023-02981-2 (PMC10129967; doi:10.1007/s00214-023-02981-2)
Supplement: Supplementary file 2 — Supplementary file2 (DOCX 28 KB) [file 214_2023_2981_MOESM2_ESM.docx]

Online Resource 1:

Article Title: Density functional theory investigation of mechanisms of degradation reactions of sulfonated PEEK membranes with OH radicals in fuel cells: Addition-elimination reactions and acid catalyzed water elimination

Journal: Theoretical Chemistry Accounts

Authors: Jonathan E. Stevens^a,b^, Courtney M. Pefley^b^, Alice Piatkowski^c^, Zachary R. Smith^d^, Nikolina Ognanovich^e^

^a^corresponding author. email: [stevenje@udmercy.edu](mailto:stevenje@udmercy.edu) phone:313-993-1048

^b^Department of Chemistry and Biochemistry, University of Detroit Mercy, Detroit MI 48221

^c^University of Detroit Mercy, School of Dentistry, Detroit MI 48208

Affiliation: Department of Chemistry and Biochemistry, University of Detroit Mercy, Detroit, MI 48221

Content

1. Table of C-H internuclear distance and energy relative to PEC-1 for stuctures obtained from constrained optimizations described in section 3-e-ii of the article.
2. Z-matrices for structures obtained from constrained optimizations described in section 3-e-ii of the article, in angstroms and degrees. The C-H internuclear distance is indicated for each Z-matrix, and the position of the constrained distance is indicated within the Z-matrix in bold.

1. Table of C-H internuclear distance and energy relative to PEC-1 for stuctures obtained from constrained optimizations described in section 3-e-ii of the article.

| C-H internuclear distance | Electronic energy relative to **PEC-1** (kcal/mol) |
| --- | --- |
| ***1.66811** | **0** |
| 1.66235 | 0.003345 |
| 1.65534 | 0.00593 |
| 1.65385 | 0.007028 |
| 1.65235 | 0.007254 |
| 1.65000 | 0.007467 |
| 1.64700 | -0.02046 |
| 1.64500 | -0.02999 |
| 1.64235 | -0.05685 |
| 1.54235 | -0.15462 |
| 1.44235 | -0.98198 |
| 1.34235 | -5.67464 |
| 1.24235 | -15.4494 |
| 1.14235 | -20.7398 |
| ****1.09569** | **-21.7363** |

*corresponds to optimized structure PEC-1

** corresponds to optimized structure PEX-1

2. Z-matrices for structures obtained from constrained optimizations described in section 3-e-ii of the article, in angstroms and degrees. The C-H internuclear distance is indicated for each Z-matrix, and the position of the constrained distance is indicated within the Z-matrix in bold.

**C-H distance:1.14235**

C

C 1 B1

C 2 B2 1 A2

C 3 B3 2 A3 1 D3

C 4 B4 3 A4 2 D4

C 1 B5 2 A5 3 D5

O 3 B6 2 A6 1 D6

O 6 B7 1 A7 2 D7

C 8 B8 6 A8 1 D8

C 7 B9 3 A9 2 D9

H 9 B10 8 A10 6 D10

H 9 B11 8 A11 6 D11

H 9 B12 8 A12 6 D12

H 10 B13 7 A13 3 D13

H 10 B14 7 A14 3 D14

H 10 B15 7 A15 3 D15

S 1 B16 2 A16 3 D16

O 17 B17 1 A17 2 D17

O 17 B18 1 A18 2 D18

O 17 B19 1 A19 2 D19

H 20 B20 17 A20 1 D20

H 2 B21 1 A21 3 D21

O 4 B22 3 A22 2 D22

H 23 B23 4 A23 3 D23

H 5 B24 4 A24 3 D24

H 4 B25 3 A25 2 D25

H 5 B26 4 A26 3 D26

O 27 B27 5 A27 4 D27

H 28 B28 27 A28 5 D28

H 28 B29 27 A29 5 D29

Variables:

B1 1.39011

B2 1.38929

A2 120.82505

B3 1.50792

A3 120.63050

D3 3.74883

B4 1.52619

A4 111.63549

D4 331.83261

B5 1.39719

A5 121.70368

D5 9.56863

B6 1.29808

A6 117.19178

D6 177.89735

B7 1.29149

A7 117.64713

D7 180.07476

B8 1.44634

A8 120.62227

D8 189.16406

B9 1.44392

A9 120.71058

D9 183.88149

B10 1.08590

A10 108.88456

D10 53.67236

B11 1.08594

A11 109.46946

D11 290.82249

B12 1.08219

A12 104.79130

D12 172.29558

B13 1.08585

A13 109.72559

D13 51.44064

B14 1.08218

A14 104.94053

D14 170.56377

B15 1.08651

A15 109.28868

D15 288.88259

B16 1.77054

A16 118.70327

D16 187.34905

B17 1.42826

A17 107.52104

D17 348.59027

B18 1.43110

A18 109.31479

D18 122.28345

B19 1.58796

A19 102.77219

D19 237.23348

B20 0.97071

A20 109.71550

D20 271.10726

B21 1.08064

A21 120.09964

D21 177.50495

B22 1.41535

A22 104.04778

D22 93.18608

B23 0.96307

A23 109.17900

D23 160.10198

B24 1.08705

A24 109.02932

D24 165.13090

B25 1.08914

A25 109.77580

D25 212.26190

**B26 1.14235**

A26 109.88896

D26 282.34677

B27 2.48945

A27 117.92326

D27 130.09126

B28 0.96194

A28 135.39362

D28 302.12658

B29 0.96096

A29 100.80484

D29 177.08263

**C-H distance: 1.24325**

C

C 1 B1

C 2 B2 1 A2

C 3 B3 2 A3 1 D3

C 4 B4 3 A4 2 D4

C 1 B5 2 A5 3 D5

O 3 B6 2 A6 1 D6

O 6 B7 1 A7 2 D7

C 8 B8 6 A8 1 D8

C 7 B9 3 A9 2 D9

H 9 B10 8 A10 6 D10

H 9 B11 8 A11 6 D11

H 9 B12 8 A12 6 D12

H 10 B13 7 A13 3 D13

H 10 B14 7 A14 3 D14

H 10 B15 7 A15 3 D15

S 1 B16 2 A16 3 D16

O 17 B17 1 A17 2 D17

O 17 B18 1 A18 2 D18

O 17 B19 1 A19 2 D19

H 20 B20 17 A20 1 D20

H 2 B21 1 A21 3 D21

O 4 B22 3 A22 2 D22

H 23 B23 4 A23 3 D23

H 5 B24 4 A24 3 D24

H 4 B25 3 A25 2 D25

H 5 B26 4 A26 3 D26

O 27 B27 5 A27 4 D27

H 28 B28 27 A28 5 D28

H 28 B29 27 A29 5 D29

Variables:

B1 1.38957

B2 1.38945

A2 120.87721

B3 1.50787

A3 120.65518

D3 4.24092

B4 1.52330

A4 111.56159

D4 332.05549

B5 1.39830

A5 121.66702

D5 9.26920

B6 1.29819

A6 117.18748

D6 178.23640

B7 1.29210

A7 117.59782

D7 179.19729

B8 1.44596

A8 120.58601

D8 189.23180

B9 1.44386

A9 120.71641

D9 183.90349

B10 1.08612

A10 108.84086

D10 54.21970

B11 1.08597

A11 109.52248

D11 291.39762

B12 1.08217

A12 104.80611

D12 172.83594

B13 1.08586

A13 109.72446

D13 51.63157

B14 1.08218

A14 104.94022

D14 170.74851

B15 1.08653

A15 109.30781

D15 289.06569

B16 1.77026

A16 118.73718

D16 186.84856

B17 1.42825

A17 107.54902

D17 349.16796

B18 1.43107

A18 109.28211

D18 122.86166

B19 1.58801

A19 102.80607

D19 237.82009

B20 0.97078

A20 109.71444

D20 271.76674

B21 1.08065

A21 120.08603

D21 177.78197

B22 1.41603

A22 104.07029

D22 93.13604

B23 0.96305

A23 109.19598

D23 159.44522

B24 1.08588

A24 109.96404

D24 166.79085

B25 1.08884

A25 109.86488

D25 212.22967

**B26 1.24235**

A26 109.50993

D26 283.43293

B27 2.45657

A27 117.74562

D27 130.02480

B28 0.96196

A28 134.57996

D28 300.14389

B29 0.96098

A29 101.01909

D29 175.59271

**C-H distance:1.34235**

C

C 1 B1

C 2 B2 1 A2

C 3 B3 2 A3 1 D3

C 4 B4 3 A4 2 D4

C 1 B5 2 A5 3 D5

O 3 B6 2 A6 1 D6

O 6 B7 1 A7 2 D7

C 8 B8 6 A8 1 D8

C 7 B9 3 A9 2 D9

H 9 B10 8 A10 6 D10

H 9 B11 8 A11 6 D11

H 9 B12 8 A12 6 D12

H 10 B13 7 A13 3 D13

H 10 B14 7 A14 3 D14

H 10 B15 7 A15 3 D15

S 1 B16 2 A16 3 D16

O 17 B17 1 A17 2 D17

O 17 B18 1 A18 2 D18

O 17 B19 1 A19 2 D19

H 20 B20 17 A20 1 D20

H 2 B21 1 A21 3 D21

O 4 B22 3 A22 2 D22

H 23 B23 4 A23 3 D23

H 5 B24 4 A24 3 D24

H 4 B25 3 A25 2 D25

H 5 B26 4 A26 3 D26

O 27 B27 5 A27 4 D27

H 28 B28 27 A28 5 D28

H 28 B29 27 A29 5 D29

Variables:

B1 1.38801

B2 1.38929

A2 120.87138

B3 1.50731

A3 120.79979

D3 5.30503

B4 1.51905

A4 111.47318

D4 332.70678

B5 1.40420

A5 121.34924

D5 8.03471

B6 1.30125

A6 117.08206

D6 179.08043

B7 1.29856

A7 116.89250

D7 177.41751

B8 1.44462

A8 120.54871

D8 182.92408

B9 1.44256

A9 120.60203

D9 183.32862

B10 1.08646

A10 109.59284

D10 57.02032

B11 1.08602

A11 109.43525

D11 294.63332

B12 1.08204

A12 104.90182

D12 175.95235

B13 1.08586

A13 109.79243

D13 52.49365

B14 1.08233

A14 104.99656

D14 171.67250

B15 1.08672

A15 109.33764

D15 289.96940

B16 1.76850

A16 118.73484

D16 186.82930

B17 1.42926

A17 107.57024

D17 350.64002

B18 1.43206

A18 109.08642

D18 123.81377

B19 1.58315

A19 103.29992

D19 239.25497

B20 0.97015

A20 109.94775

D20 275.43490

B21 1.08060

A21 120.09458

D21 178.20805

B22 1.41976

A22 104.09841

D22 94.34515

B23 0.96296

A23 109.02053

D23 160.11651

B24 1.08496

A24 111.15421

D24 168.84439

B25 1.08918

A25 109.77273

D25 212.78281

**B26 1.34235**

A26 108.13997

D26 284.45743

B27 1.71034

A27 174.35578

D27 81.92866

B28 0.96263

A28 110.31465

D28 250.33536

B29 0.96275

A29 110.48922

D29 134.22579

**C-H distance: 1.44235**

C

C 1 B1

C 2 B2 1 A2

C 3 B3 2 A3 1 D3

C 4 B4 3 A4 2 D4

C 5 B5 4 A5 3 D5

O 3 B6 2 A6 1 D6

O 6 B7 5 A7 4 D7

C 8 B8 6 A8 5 D8

C 7 B9 3 A9 2 D9

H 9 B10 8 A10 6 D10

H 9 B11 8 A11 6 D11

H 9 B12 8 A12 6 D12

H 10 B13 7 A13 3 D13

H 10 B14 7 A14 3 D14

H 10 B15 7 A15 3 D15

S 1 B16 2 A16 3 D16

O 17 B17 1 A17 2 D17

O 17 B18 1 A18 2 D18

O 17 B19 1 A19 2 D19

H 20 B20 17 A20 1 D20

H 2 B21 1 A21 3 D21

O 4 B22 3 A22 2 D22

H 23 B23 4 A23 3 D23

H 5 B24 4 A24 3 D24

H 4 B25 3 A25 2 D25

H 5 B26 4 A26 3 D26

O 27 B27 5 A27 4 D27

H 28 B28 27 A28 5 D28

H 28 B29 27 A29 5 D29

Variables:

B1 1.38347

B2 1.38731

A2 120.80239

B3 1.50521

A3 121.13328

D3 5.35345

B4 1.51385

A4 111.33419

D4 335.53592

B5 1.40945

A5 117.89240

D5 33.38899

B6 1.31063

A6 117.01508

D6 179.60084

B7 1.31189

A7 124.38970

D7 162.41165

B8 1.43862

A8 119.61046

D8 354.01450

B9 1.43894

A9 120.39733

D9 181.48243

B10 1.08746

A10 109.81402

D10 61.57745

B11 1.08677

A11 109.73745

D11 299.33547

B12 1.08249

A12 105.19275

D12 180.45526

B13 1.08591

A13 110.06584

D13 53.42149

B14 1.08277

A14 105.12060

D14 172.64891

B15 1.08721

A15 109.57771

D15 290.86964

B16 1.75927

A16 119.06592

D16 185.68519

B17 1.43083

A17 108.00157

D17 350.05330

B18 1.43352

A18 109.42331

D18 123.23322

B19 1.58660

A19 103.66829

D19 238.66277

B20 0.97035

A20 109.52394

D20 279.88295

B21 1.08056

A21 120.18962

D21 178.80760

B22 1.42635

A22 104.62314

D22 97.81959

B23 0.96291

A23 108.70747

D23 164.16742

B24 1.08330

A24 113.65554

D24 175.76764

B25 1.09098

A25 109.56432

D25 215.19355

**B26 1.44235**

A26 103.75289

D26 286.20206

B27 1.27416

A27 171.98822

D27 344.48952

B28 0.96749

A28 110.28302

D28 280.89337

B29 0.96760

A29 111.00936

D29 162.36841

**C-H distance: 1.54235**

C

C 1 B1

C 2 B2 1 A2

C 3 B3 2 A3 1 D3

C 4 B4 3 A4 2 D4

C 5 B5 4 A5 3 D5

O 3 B6 2 A6 1 D6

O 6 B7 5 A7 4 D7

C 8 B8 6 A8 5 D8

C 7 B9 3 A9 2 D9

H 9 B10 8 A10 6 D10

H 9 B11 8 A11 6 D11

H 9 B12 8 A12 6 D12

H 10 B13 7 A13 3 D13

H 10 B14 7 A14 3 D14

H 10 B15 7 A15 3 D15

S 1 B16 2 A16 3 D16

O 17 B17 1 A17 2 D17

O 17 B18 1 A18 2 D18

O 17 B19 1 A19 2 D19

H 20 B20 17 A20 1 D20

H 2 B21 1 A21 3 D21

O 4 B22 3 A22 2 D22

H 23 B23 4 A23 3 D23

H 5 B24 4 A24 3 D24

H 4 B25 3 A25 2 D25

H 5 B26 4 A26 3 D26

O 27 B27 5 A27 4 D27

H 28 B28 27 A28 5 D28

H 28 B29 27 A29 5 D29

Variables:

B1 1.38144

B2 1.38588

A2 120.74066

B3 1.50420

A3 121.49707

D3 5.57663

B4 1.51076

A4 111.72066

D4 339.16593

B5 1.38486

A5 119.62166

D5 27.87836

B6 1.31611

A6 117.00560

D6 180.25750

B7 1.32349

A7 125.01417

D7 165.55086

B8 1.43471

A8 119.08931

D8 352.00118

B9 1.43708

A9 120.34723

D9 179.00453

B10 1.08797

A10 110.15779

D10 64.65889

B11 1.08739

A11 109.85131

D11 302.40741

B12 1.08286

A12 105.37727

D12 183.47266

B13 1.08583

A13 110.25465

D13 54.74917

B14 1.08301

A14 105.17545

D14 174.00936

B15 1.08763

A15 109.68610

D15 292.15079

B16 1.75516

A16 119.15475

D16 184.83700

B17 1.43174

A17 108.32008

D17 348.42280

B18 1.43437

A18 109.60146

D18 121.58735

B19 1.58794

A19 103.70606

D19 237.04286

B20 0.97089

A20 109.21638

D20 284.48369

B21 1.08051

A21 120.24923

D21 179.67337

B22 1.42939

A22 105.03767

D22 101.16470

B23 0.96292

A23 108.59634

D23 164.05180

B24 1.08255

A24 114.83559

D24 178.54271

B25 1.09191

A25 109.41573

D25 218.14965

**B26 1.54235**

A26 102.41822

D26 283.55931

B27 1.13834

A27 170.63606

D27 318.10425

B28 0.97066

A28 110.26796

D28 275.14438

B29 0.97066

A29 111.04670

D29 155.11035

**C-H distance:1.64235**

C

C 1 B1

C 2 B2 1 A2

C 3 B3 2 A3 1 D3

C 4 B4 3 A4 2 D4

C 5 B5 4 A5 3 D5

O 3 B6 2 A6 1 D6

O 6 B7 5 A7 4 D7

C 8 B8 6 A8 5 D8

C 7 B9 3 A9 2 D9

H 9 B10 8 A10 6 D10

H 9 B11 8 A11 6 D11

H 9 B12 8 A12 6 D12

H 10 B13 7 A13 3 D13

H 10 B14 7 A14 3 D14

H 10 B15 7 A15 3 D15

S 1 B16 2 A16 3 D16

O 17 B17 1 A17 2 D17

O 17 B18 1 A18 2 D18

O 17 B19 1 A19 2 D19

H 20 B20 17 A20 1 D20

H 2 B21 1 A21 3 D21

O 4 B22 3 A22 2 D22

H 23 B23 4 A23 3 D23

H 5 B24 4 A24 3 D24

H 4 B25 3 A25 2 D25

H 5 B26 4 A26 3 D26

O 27 B27 5 A27 4 D27

H 28 B28 27 A28 5 D28

H 28 B29 27 A29 5 D29

Variables:

B1 1.38062

B2 1.38542

A2 120.64373

B3 1.50382

A3 121.58288

D3 5.14753

B4 1.50892

A4 111.82102

D4 340.30858

B5 1.37450

A5 120.23221

D5 26.07293

B6 1.31849

A6 117.04313

D6 180.13412

B7 1.33042

A7 125.27618

D7 167.23380

B8 1.43298

A8 118.77710

D8 352.06072

B9 1.43625

A9 120.28616

D9 178.12607

B10 1.08856

A10 110.26805

D10 65.91410

B11 1.08766

A11 109.94104

D11 303.65912

B12 1.08306

A12 105.46643

D12 184.68986

B13 1.08589

A13 110.33152

D13 54.97963

B14 1.08310

A14 105.20973

D14 174.24176

B15 1.08783

A15 109.73451

D15 292.36044

B16 1.75334

A16 119.20376

D16 184.92960

B17 1.43210

A17 108.48889

D17 348.54299

B18 1.43476

A18 109.60325

D18 121.68878

B19 1.58845

A19 103.74603

D19 237.14554

B20 0.97122

A20 109.03385

D20 286.68670

B21 1.08046

A21 120.29981

D21 179.47162

B22 1.43096

A22 105.24866

D22 102.08384

B23 0.96301

A23 108.51512

D23 164.86984

B24 1.08147

A24 115.61225

D24 182.28751

B25 1.09226

A25 109.46551

D25 218.85591

**B26 1.64235**

A26 101.59376

D26 287.15006

B27 1.08636

A27 171.81093

D27 336.41276

B28 0.97204

A28 110.38935

D28 296.05503

B29 0.97213

A29 110.95847

D29 175.59055

**C-H distance:1.65235**

C

C 1 B1

C 2 B2 1 A2

C 3 B3 2 A3 1 D3

C 4 B4 3 A4 2 D4

C 5 B5 4 A5 3 D5

O 3 B6 2 A6 1 D6

O 6 B7 5 A7 4 D7

C 8 B8 6 A8 5 D8

C 7 B9 3 A9 2 D9

H 9 B10 8 A10 6 D10

H 9 B11 8 A11 6 D11

H 9 B12 8 A12 6 D12

H 10 B13 7 A13 3 D13

H 10 B14 7 A14 3 D14

H 10 B15 7 A15 3 D15

S 1 B16 2 A16 3 D16

O 17 B17 1 A17 2 D17

O 17 B18 1 A18 2 D18

O 17 B19 1 A19 2 D19

H 20 B20 17 A20 1 D20

H 2 B21 1 A21 3 D21

O 4 B22 3 A22 2 D22

H 23 B23 4 A23 3 D23

H 5 B24 4 A24 3 D24

H 4 B25 3 A25 2 D25

H 5 B26 4 A26 3 D26

O 27 B27 5 A27 4 D27

H 28 B28 27 A28 5 D28

H 28 B29 27 A29 5 D29

Variables:

B1 1.38061

B2 1.38512

A2 120.65593

B3 1.50366

A3 121.65879

D3 5.49401

B4 1.50893

A4 111.95947

D4 341.02297

B5 1.37410

A5 120.26803

D5 25.13992

B6 1.31881

A6 117.02397

D6 180.49418

B7 1.33109

A7 125.25893

D7 166.85763

B8 1.43290

A8 118.72093

D8 350.32529

B9 1.43613

A9 120.30764

D9 177.81564

B10 1.08826

A10 110.39754

D10 66.09356

B11 1.08774

A11 109.84989

D11 303.90512

B12 1.08308

A12 105.48134

D12 184.92551

B13 1.08584

A13 110.34155

D13 55.34667

B14 1.08312

A14 105.20752

D14 174.62007

B15 1.08786

A15 109.74887

D15 292.73061

B16 1.75332

A16 119.17877

D16 184.65218

B17 1.43218

A17 108.48324

D17 347.30795

B18 1.43484

A18 109.69148

D18 120.45522

B19 1.58843

A19 103.68425

D19 235.91096

B20 0.97128

A20 109.02639

D20 286.75506

B21 1.08046

A21 120.30365

D21 180.02532

B22 1.43135

A22 105.24122

D22 102.64148

B23 0.96301

A23 108.52650

D23 164.45380

B24 1.08161

A24 115.46574

D24 180.99637

B25 1.09215

A25 109.40557

D25 219.32728

**B26 1.65235**

A26 102.84089

D26 285.43409

B27 1.08371

A27 173.55856

D27 330.05633

B28 0.97218

A28 110.41464

D28 316.82257

B29 0.97237

A29 110.62659

D29 196.70602

**C-H distance:1.64500**

C

C 1 B1

C 2 B2 1 A2

C 3 B3 2 A3 1 D3

C 4 B4 3 A4 2 D4

C 5 B5 4 A5 3 D5

O 3 B6 2 A6 1 D6

O 6 B7 5 A7 4 D7

C 8 B8 6 A8 5 D8

C 7 B9 3 A9 2 D9

H 9 B10 8 A10 6 D10

H 9 B11 8 A11 6 D11

H 9 B12 8 A12 6 D12

H 10 B13 7 A13 3 D13

H 10 B14 7 A14 3 D14

H 10 B15 7 A15 3 D15

S 1 B16 2 A16 3 D16

O 17 B17 1 A17 2 D17

O 17 B18 1 A18 2 D18

O 17 B19 1 A19 2 D19

H 20 B20 17 A20 1 D20

H 2 B21 1 A21 3 D21

O 4 B22 3 A22 2 D22

H 23 B23 4 A23 3 D23

H 5 B24 4 A24 3 D24

H 4 B25 3 A25 2 D25

H 5 B26 4 A26 3 D26

O 27 B27 5 A27 4 D27

H 28 B28 27 A28 5 D28

H 28 B29 27 A29 5 D29

Variables:

B1 1.38061

B2 1.38540

A2 120.64360

B3 1.50380

A3 121.58612

D3 5.15711

B4 1.50888

A4 111.82666

D4 340.34635

B5 1.37429

A5 120.25046

D5 25.99458

B6 1.31855

A6 117.04340

D6 180.14643

B7 1.33057

A7 125.28007

D7 167.28382

B8 1.43293

A8 118.76904

D8 352.06715

B9 1.43623

A9 120.28506

D9 178.10020

B10 1.08857

A10 110.27130

D10 65.91460

B11 1.08766

A11 109.94201

D11 303.66153

B12 1.08307

A12 105.46904

D12 184.69130

B13 1.08589

A13 110.33324

D13 54.99838

B14 1.08311

A14 105.21033

D14 174.26105

B15 1.08783

A15 109.73594

D15 292.37919

B16 1.75329

A16 119.20521

D16 184.91771

B17 1.43211

A17 108.49291

D17 348.53206

B18 1.43477

A18 109.60485

D18 121.67797

B19 1.58846

A19 103.74528

D19 237.13443

B20 0.97122

A20 109.02975

D20 286.73399

B21 1.08046

A21 120.29992

D21 179.48307

B22 1.43099

A22 105.25351

D22 102.11827

B23 0.96301

A23 108.51363

D23 164.86725

B24 1.08146

A24 115.62169

D24 182.32310

B25 1.09226

A25 109.46335

D25 218.88705

**B26 1.64500**

A26 101.57718

D26 287.12618

B27 1.08530

A27 171.77635

D27 336.41084

B28 0.97207

A28 110.38876

D28 295.97082

B29 0.97215

A29 110.96157

D29 175.48732

**C-H distance:1.64700**

C

C 1 B1

C 2 B2 1 A2

C 3 B3 2 A3 1 D3

C 4 B4 3 A4 2 D4

C 5 B5 4 A5 3 D5

O 3 B6 2 A6 1 D6

O 6 B7 5 A7 4 D7

C 8 B8 6 A8 5 D8

C 7 B9 3 A9 2 D9

H 9 B10 8 A10 6 D10

H 9 B11 8 A11 6 D11

H 9 B12 8 A12 6 D12

H 10 B13 7 A13 3 D13

H 10 B14 7 A14 3 D14

H 10 B15 7 A15 3 D15

S 1 B16 2 A16 3 D16

O 17 B17 1 A17 2 D17

O 17 B18 1 A18 2 D18

O 17 B19 1 A19 2 D19

H 20 B20 17 A20 1 D20

H 2 B21 1 A21 3 D21

O 4 B22 3 A22 2 D22

H 23 B23 4 A23 3 D23

H 5 B24 4 A24 3 D24

H 4 B25 3 A25 2 D25

H 5 B26 4 A26 3 D26

O 27 B27 5 A27 4 D27

H 28 B28 27 A28 5 D28

H 28 B29 27 A29 5 D29

Variables:

B1 1.38059

B2 1.38538

A2 120.64312

B3 1.50379

A3 121.58825

D3 5.16370

B4 1.50884

A4 111.83028

D4 340.37084

B5 1.37414

A5 120.26223

D5 25.94390

B6 1.31859

A6 117.04336

D6 180.15398

B7 1.33069

A7 125.28323

D7 167.31154

B8 1.43290

A8 118.76368

D8 352.05036

B9 1.43622

A9 120.28435

D9 178.08247

B10 1.08858

A10 110.27541

D10 65.92985

B11 1.08767

A11 109.94177

D11 303.67742

B12 1.08307

A12 105.47066

D12 184.70706

B13 1.08589

A13 110.33463

D13 55.00803

B14 1.08311

A14 105.21080

D14 174.27082

B15 1.08784

A15 109.73694

D15 292.38842

B16 1.75326

A16 119.20625

D16 184.91189

B17 1.43211

A17 108.49565

D17 348.51956

B18 1.43478

A18 109.60627

D18 121.66535

B19 1.58847

A19 103.74462

D19 237.12153

B20 0.97123

A20 109.02668

D20 286.76887

B21 1.08046

A21 120.30032

D21 179.49017

B22 1.43102

A22 105.25612

D22 102.13865

B23 0.96301

A23 108.51343

D23 164.86792

B24 1.08145

A24 115.62987

D24 182.35788

B25 1.09226

A25 109.46301

D25 218.90510

**B26 1.64700**

A26 101.57304

D26 287.13116

B27 1.08452

A27 171.76472

D27 336.18208

B28 0.97209

A28 110.38757

D28 296.26324

B29 0.97217

A29 110.96224

D29 175.76885

**C-H distance:1.65000**

C

C 1 B1

C 2 B2 1 A2

C 3 B3 2 A3 1 D3

C 4 B4 3 A4 2 D4

C 5 B5 4 A5 3 D5

O 3 B6 2 A6 1 D6

O 6 B7 5 A7 4 D7

C 8 B8 6 A8 5 D8

C 7 B9 3 A9 2 D9

H 9 B10 8 A10 6 D10

H 9 B11 8 A11 6 D11

H 9 B12 8 A12 6 D12

H 10 B13 7 A13 3 D13

H 10 B14 7 A14 3 D14

H 10 B15 7 A15 3 D15

S 1 B16 2 A16 3 D16

O 17 B17 1 A17 2 D17

O 17 B18 1 A18 2 D18

O 17 B19 1 A19 2 D19

H 20 B20 17 A20 1 D20

H 2 B21 1 A21 3 D21

O 4 B22 3 A22 2 D22

H 23 B23 4 A23 3 D23

H 5 B24 4 A24 3 D24

H 4 B25 3 A25 2 D25

H 5 B26 4 A26 3 D26

O 27 B27 5 A27 4 D27

H 28 B28 27 A28 5 D28

H 28 B29 27 A29 5 D29

Variables:

B1 1.38060

B2 1.38511

A2 120.65580

B3 1.50367

A3 121.64952

D3 5.50373

B4 1.50899

A4 111.95172

D4 340.94457

B5 1.37434

A5 120.25115

D5 25.25845

B6 1.31876

A6 117.02339

D6 180.47863

B7 1.33087

A7 125.26485

D7 166.78744

B8 1.43295

A8 118.75019

D8 350.43560

B9 1.43615

A9 120.30988

D9 177.85904

B10 1.08825

A10 110.39547

D10 66.13677

B11 1.08773

A11 109.85688

D11 303.93654

B12 1.08307

A12 105.47681

D12 184.96222

B13 1.08584

A13 110.34043

D13 55.32419

B14 1.08312

A14 105.20739

D14 174.59519

B15 1.08785

A15 109.74734

D15 292.70712

B16 1.75336

A16 119.17345

D16 184.67343

B17 1.43217

A17 108.47833

D17 347.33900

B18 1.43483

A18 109.69050

D18 120.48803

B19 1.58842

A19 103.68681

D19 235.94525

B20 0.97125

A20 109.03210

D20 286.63092

B21 1.08046

A21 120.30333

D21 179.99730

B22 1.43130

A22 105.23700

D22 102.56667

B23 0.96301

A23 108.52682

D23 164.43946

B24 1.08162

A24 115.45373

D24 180.97023

B25 1.09212

A25 109.40536

D25 219.25627

**B26 1.65000**

A26 102.80232

D26 285.45881

B27 1.08474

A27 173.49651

D27 332.08028

B28 0.97216

A28 110.42107

D28 314.69780

B29 0.97232

A29 110.64061

D29 194.58628

**C-H distance:1.65235**

C

C 1 B1

C 2 B2 1 A2

C 3 B3 2 A3 1 D3

C 4 B4 3 A4 2 D4

C 5 B5 4 A5 3 D5

O 3 B6 2 A6 1 D6

O 6 B7 5 A7 4 D7

C 8 B8 6 A8 5 D8

C 7 B9 3 A9 2 D9

H 9 B10 8 A10 6 D10

H 9 B11 8 A11 6 D11

H 9 B12 8 A12 6 D12

H 10 B13 7 A13 3 D13

H 10 B14 7 A14 3 D14

H 10 B15 7 A15 3 D15

S 1 B16 2 A16 3 D16

O 17 B17 1 A17 2 D17

O 17 B18 1 A18 2 D18

O 17 B19 1 A19 2 D19

H 20 B20 17 A20 1 D20

H 2 B21 1 A21 3 D21

O 4 B22 3 A22 2 D22

H 23 B23 4 A23 3 D23

H 5 B24 4 A24 3 D24

H 4 B25 3 A25 2 D25

H 5 B26 4 A26 3 D26

O 27 B27 5 A27 4 D27

H 28 B28 27 A28 5 D28

H 28 B29 27 A29 5 D29

Variables:

B1 1.38061

B2 1.38512

A2 120.65593

B3 1.50366

A3 121.65879

D3 5.49401

B4 1.50893

A4 111.95947

D4 341.02297

B5 1.37410

A5 120.26803

D5 25.13992

B6 1.31881

A6 117.02397

D6 180.49418

B7 1.33109

A7 125.25893

D7 166.85763

B8 1.43290

A8 118.72093

D8 350.32529

B9 1.43613

A9 120.30764

D9 177.81564

B10 1.08826

A10 110.39754

D10 66.09356

B11 1.08774

A11 109.84989

D11 303.90512

B12 1.08308

A12 105.48134

D12 184.92551

B13 1.08584

A13 110.34155

D13 55.34667

B14 1.08312

A14 105.20752

D14 174.62007

B15 1.08786

A15 109.74887

D15 292.73061

B16 1.75332

A16 119.17877

D16 184.65218

B17 1.43218

A17 108.48324

D17 347.30795

B18 1.43484

A18 109.69148

D18 120.45522

B19 1.58843

A19 103.68425

D19 235.91096

B20 0.97128

A20 109.02639

D20 286.75506

B21 1.08046

A21 120.30365

D21 180.02532

B22 1.43135

A22 105.24122

D22 102.64148

B23 0.96301

A23 108.52650

D23 164.45380

B24 1.08161

A24 115.46574

D24 180.99637

B25 1.09215

A25 109.40557

D25 219.32728

**B26 1.65235**

A26 102.84089

D26 285.43409

B27 1.08371

A27 173.55856

D27 330.05633

B28 0.97218

A28 110.41464

D28 316.82257

B29 0.97237

A29 110.62659

D29 196.70602

**C-H distance:1.65385**

C

C 1 B1

C 2 B2 1 A2

C 3 B3 2 A3 1 D3

C 4 B4 3 A4 2 D4

C 5 B5 4 A5 3 D5

O 3 B6 2 A6 1 D6

O 6 B7 5 A7 4 D7

C 8 B8 6 A8 5 D8

C 7 B9 3 A9 2 D9

H 9 B10 8 A10 6 D10

H 9 B11 8 A11 6 D11

H 9 B12 8 A12 6 D12

H 10 B13 7 A13 3 D13

H 10 B14 7 A14 3 D14

H 10 B15 7 A15 3 D15

S 1 B16 2 A16 3 D16

O 17 B17 1 A17 2 D17

O 17 B18 1 A18 2 D18

O 17 B19 1 A19 2 D19

H 20 B20 17 A20 1 D20

H 2 B21 1 A21 3 D21

O 4 B22 3 A22 2 D22

H 23 B23 4 A23 3 D23

H 5 B24 4 A24 3 D24

H 4 B25 3 A25 2 D25

H 5 B26 4 A26 3 D26

O 27 B27 5 A27 4 D27

H 28 B28 27 A28 5 D28

H 28 B29 27 A29 5 D29

Variables:

B1 1.38058

B2 1.38508

A2 120.65667

B3 1.50363

A3 121.65479

D3 5.50790

B4 1.50890

A4 111.96095

D4 341.01719

B5 1.37404

A5 120.27795

D5 25.13558

B6 1.31885

A6 117.02388

D6 180.49518

B7 1.33110

A7 125.26738

D7 166.85461

B8 1.43289

A8 118.73202

D8 350.37802

B9 1.43613

A9 120.30902

D9 177.81091

B10 1.08825

A10 110.40283

D10 66.10038

B11 1.08774

A11 109.85354

D11 303.90911

B12 1.08307

A12 105.48198

D12 184.93104

B13 1.08584

A13 110.34492

D13 55.31111

B14 1.08312

A14 105.20828

D14 174.58281

B15 1.08786

A15 109.74710

D15 292.69347

B16 1.75329

A16 119.17709

D16 184.65350

B17 1.43218

A17 108.48357

D17 347.31131

B18 1.43485

A18 109.69425

D18 120.46105

B19 1.58844

A19 103.68438

D19 235.91711

B20 0.97127

A20 109.02651

D20 286.69309

B21 1.08046

A21 120.30325

D21 180.01436

B22 1.43135

A22 105.24572

D22 102.63554

B23 0.96301

A23 108.52576

D23 164.44630

B24 1.08160

A24 115.46767

D24 181.02317

B25 1.09214

A25 109.40093

D25 219.32392

**B26 1.65385**

A26 102.80172

D26 285.44885

B27 1.08326

A27 173.50830

D27 331.18253

B28 0.97220

A28 110.41413

D28 315.67582

B29 0.97237

A29 110.63818

D29 195.54767

**C-H distance:1.65534**

C

C 1 B1

C 2 B2 1 A2

C 3 B3 2 A3 1 D3

C 4 B4 3 A4 2 D4

C 5 B5 4 A5 3 D5

O 3 B6 2 A6 1 D6

O 6 B7 5 A7 4 D7

C 8 B8 6 A8 5 D8

C 7 B9 3 A9 2 D9

H 9 B10 8 A10 6 D10

H 9 B11 8 A11 6 D11

H 9 B12 8 A12 6 D12

H 10 B13 7 A13 3 D13

H 10 B14 7 A14 3 D14

H 10 B15 7 A15 3 D15

S 1 B16 2 A16 3 D16

O 17 B17 1 A17 2 D17

O 17 B18 1 A18 2 D18

O 17 B19 1 A19 2 D19

H 20 B20 17 A20 1 D20

H 2 B21 1 A21 3 D21

O 4 B22 3 A22 2 D22

H 23 B23 4 A23 3 D23

H 5 B24 4 A24 3 D24

H 4 B25 3 A25 2 D25

H 5 B26 4 A26 3 D26

O 27 B27 5 A27 4 D27

H 28 B28 27 A28 5 D28

H 28 B29 27 A29 5 D29

Variables:

B1 1.38058

B2 1.38506

A2 120.65764

B3 1.50362

A3 121.66244

D3 5.45830

B4 1.50880

A4 111.96733

D4 341.10087

B5 1.37380

A5 120.29943

D5 25.04069

B6 1.31891

A6 117.02139

D6 180.45457

B7 1.33129

A7 125.26240

D7 166.91205

B8 1.43286

A8 118.70979

D8 350.24646

B9 1.43611

A9 120.31088

D9 177.78868

B10 1.08825

A10 110.41467

D10 66.03797

B11 1.08776

A11 109.84535

D11 303.86286

B12 1.08308

A12 105.48745

D12 184.88016

B13 1.08583

A13 110.34634

D13 55.33174

B14 1.08313

A14 105.20833

D14 174.60391

B15 1.08786

A15 109.74910

D15 292.71325

B16 1.75322

A16 119.18507

D16 184.65132

B17 1.43219

A17 108.48846

D17 347.33532

B18 1.43485

A18 109.69804

D18 120.48673

B19 1.58847

A19 103.68110

D19 235.94038

B20 0.97128

A20 109.02182

D20 286.75356

B21 1.08046

A21 120.30348

D21 179.98380

B22 1.43141

A22 105.25264

D22 102.70841

B23 0.96301

A23 108.52609

D23 164.43394

B24 1.08158

A24 115.47502

D24 181.05702

B25 1.09215

A25 109.40055

D25 219.39889

**B26 1.65534**

A26 102.83749

D26 285.44055

B27 1.08212

A27 173.62103

D27 329.08479

B28 0.97223

A28 110.40271

D28 318.23162

B29 0.97243

A29 110.61739

D29 198.10279

**C-H distance:1.66235**

C

C 1 B1

C 2 B2 1 A2

C 3 B3 2 A3 1 D3

C 4 B4 3 A4 2 D4

C 5 B5 4 A5 3 D5

O 3 B6 2 A6 1 D6

O 6 B7 5 A7 4 D7

C 8 B8 6 A8 5 D8

C 7 B9 3 A9 2 D9

H 9 B10 8 A10 6 D10

H 9 B11 8 A11 6 D11

H 9 B12 8 A12 6 D12

H 10 B13 7 A13 3 D13

H 10 B14 7 A14 3 D14

H 10 B15 7 A15 3 D15

S 1 B16 2 A16 3 D16

O 17 B17 1 A17 2 D17

O 17 B18 1 A18 2 D18

O 17 B19 1 A19 2 D19

H 20 B20 17 A20 1 D20

H 2 B21 1 A21 3 D21

O 4 B22 3 A22 2 D22

H 23 B23 4 A23 3 D23

H 5 B24 4 A24 3 D24

H 4 B25 3 A25 2 D25

H 5 B26 4 A26 3 D26

O 27 B27 5 A27 4 D27

H 28 B28 27 A28 5 D28

H 28 B29 27 A29 5 D29

Variables:

B1 1.38055

B2 1.38501

A2 120.65815

B3 1.50358

A3 121.66922

D3 5.53907

B4 1.50872

A4 111.97977

D4 341.17467

B5 1.37339

A5 120.33982

D5 24.84747

B6 1.31904

A6 117.02244

D6 180.53886

B7 1.33158

A7 125.27221

D7 167.00862

B8 1.43276

A8 118.69884

D8 350.29164

B9 1.43606

A9 120.30686

D9 177.70799

B10 1.08827

A10 110.41729

D10 66.08303

B11 1.08777

A11 109.85069

D11 303.90623

B12 1.08308

A12 105.49173

D12 184.92318

B13 1.08584

A13 110.34912

D13 55.42721

B14 1.08313

A14 105.20974

D14 174.70108

B15 1.08787

A15 109.75420

D15 292.80965

B16 1.75314

A16 119.18300

D16 184.61430

B17 1.43221

A17 108.49547

D17 347.22696

B18 1.43488

A18 109.70155

D18 120.37656

B19 1.58848

A19 103.68023

D19 235.83100

B20 0.97130

A20 109.01403

D20 286.83945

B21 1.08046

A21 120.30332

D21 180.06477

B22 1.43147

A22 105.25927

D22 102.77528

B23 0.96301

A23 108.52420

D23 164.41253

B24 1.08156

A24 115.49350

D24 181.09287

B25 1.09216

A25 109.39665

D25 219.45500

**B26 1.66235**

A26 102.79203

D26 285.34359

B27 1.08007

A27 173.54151

D27 330.29357

B28 0.97230

A28 110.40339

D28 316.93615

B29 0.97249

A29 110.61470

D29 196.78606
